# Php4 Is a Key Player for Iron Economy in Meiotic and Sporulating Cells

**DOI:** 10.1534/g3.116.031898

**Published:** 2016-07-26

**Authors:** Ariane Brault, Charalampos Rallis, Vincent Normant, Jean-Michel Garant, Jürg Bähler, Simon Labbé

**Affiliations:** * Département de Biochimie, Faculté de Médecine et des Sciences de la Santé, Université de Sherbrooke, Quebec, J1E 4K8, Canada; † Research Department of Genetics, Evolution and Environment, University College London, WC1E 6BT, UK

**Keywords:** iron-sparing response, iron-regulated genes, CCAAT-binding factor, meiosis, fission yeast

## Abstract

Meiosis is essential for sexually reproducing organisms, including the fission yeast *Schizosaccharomyces pombe*. In meiosis, chromosomes replicate once in a diploid precursor cell (zygote), and then segregate twice to generate four haploid meiotic products, named spores in yeast. In *S. pombe*, Php4 is responsible for the transcriptional repression capability of the heteromeric CCAAT-binding factor to negatively regulate genes encoding iron-using proteins under low-iron conditions. Here, we show that the CCAAT-regulatory subunit Php4 is required for normal progression of meiosis under iron-limiting conditions. Cells lacking Php4 exhibit a meiotic arrest at metaphase I. Microscopic analyses of cells expressing functional GFP-Php4 show that it colocalizes with chromosomal material at every stage of meiosis under low concentrations of iron. In contrast, GFP-Php4 fluorescence signal is lost when cells undergo meiosis under iron-replete conditions. Global gene expression analysis of meiotic cells using DNA microarrays identified 137 genes that are regulated in an iron- and Php4-dependent manner. Among them, 18 genes are expressed exclusively during meiosis and constitute new putative Php4 target genes, which include *hry1^+^* and *mug14^+^*. Further analysis validates that Php4 is required for maximal and timely repression of *hry1^+^* and *mug14^+^* genes. Using a chromatin immunoprecipitation approach, we show that Php4 specifically associates with *hry1^+^* and *mug14^+^* promoters *in vivo*. Taken together, the results reveal that in iron-starved meiotic cells, Php4 is essential for completion of the meiotic program since it participates in global gene expression reprogramming to optimize the use of limited available iron.

Eukaryotic organisms that sexually reproduce have a specialized type of cell division that enables the formation of haploid gametes from diploid germ cells. This specialized mode of cell division is called meiosis (Marston and Amon 2004; Handel and Schimenti 2010; Ohkura 2015). The early stage of meiosis involves a round of DNA synthesis during which chromosomal material is duplicated, generating pairs of homologous chromosomes. The subsequent step consists of meiotic recombination between homologous chromosomes that increases genetic diversity and the potential appearance of new phenotypic traits. This step is followed by two successive meiotic divisions (denoted meiosis I and II), in which homologous chromosomes and then sister chromatids are separated to generate four haploid sets of chromosomes that are inheritable by the next generation. In fungi, including *Schizosaccharomyces pombe*, the terminal stage of meiosis involves a differentiation program that induces compartmentalization of the genetic material into four spores (or gametes) that are enclosed into an ascus (Sabatinos and Forsburg 2010; Shigehisa *et al.* 2010).

Studies using different model organisms have shown that micronutrients, including transition metals such as zinc and copper, are required for the normal progression of meiosis (Kim *et al.* 2010; Beaudoin *et al.* 2011). In mice, zinc insufficient oocytes proceed through segregation of homologous chromosomes (meiosis I) but fail to further segregate sister chromatids, therefore blocking meiotic progression past telophase I (Kim *et al.* 2010). In addition, maturation of porcine oocytes under conditions of zinc insufficiency is blocked at metaphase I, leading to a failure to segregate homologous chromosomes (Jeon *et al.* 2015). Similarly, studies with the fission yeast *S. pombe* have revealed that copper deficiency arrests meiosis by blocking the process at metaphase I (Beaudoin *et al.* 2011). On the basis of these observations, it is reasonable to suggest that iron, one of the most used transition metals in biology, may also be required during meiotic differentiation.

*S. pombe* was used here as a model to characterize iron requirement during meiosis since it is one of the best understood model systems to investigate the eukaryotic cell cycle by way of conventional mode of division (mitosis) or meiotic cell division program (meiosis) (Navarro *et al.* 2012; Hoffman *et al.* 2015). In this context, growth conditions and temperature-sensitive strains have been developed that allow the synchronization of cells prior to their entry into the meiotic program (Mata *et al.* 2002; Harigaya and Yamamoto 2007). For instance, haploid cells arrest in the G_1_ phase of the cell cycle under low-nitrogen conditions. When cells of the opposite mating type interact during the G_1_ block, haploid cells conjugate to form diploid zygotes. If the resulting zygotes are maintained under nitrogen-starved conditions, they undergo meiosis by a process called zygotic meiosis. Alternatively, zygotes freshly formed can be returned to a nitrogen-replete medium before their commitment to meiosis and they will grow as diploids for a period of time. Over this period of time, if these diploid cells undergo a second switch from sufficient to insufficient nitrogen, their passage to meiosis occurs very rapidly and in a more synchronous manner than zygotic meiosis by a process called azygotic meiosis. Mitotically growing cells produce an active protein kinase called Pat1 that inhibits cells from entering meiosis. When active, Pat1 phosphorylates the transcription factor Ste11 and the meiosis-specific inducer Mei2. This Pat1-mediated posttranslational modification blocks their activity. A mutant strain containing a temperature-sensitive *pat1-114* allele produces a thermolabile Pat1 kinase. When *pat1-114* cells undergo a transition from low (25°) to elevated (34°) temperature, Pat1 is readily inactivated, triggering a cell cycle switch from mitosis to meiosis in a highly efficient and synchronous fashion. This latter system, termed *pat1-induced* meiosis, is more synchronous than azygotic meiosis (Yamamoto 2004; Doll *et al.* 2008).

In *S. pombe*, Fep1 and Php4 are two iron-dependent regulatory proteins that play a critical role in maintaining cellular iron homeostasis (Labbé *et al.* 2013; Brault *et al.* 2015). Their roles have traditionally been investigated in dividing cells that grow mitotically. In response to high concentrations of iron, the GATA-type transcription factor Fep1 binds to GATA elements and represses several genes encoding proteins that are involved in iron acquisition (Jbel *et al.* 2009). Fep1 also represses the expression of Php4, which is a negative iron-dependent regulatory subunit of the heteromeric CCAAT-binding factor (Mercier *et al.* 2006). In contrast, when iron levels are low, Fep1 becomes inactive and loses its ability to interact with chromatin. This situation leads to transcriptional activation of the Fep1 regulon, which includes the *php4^+^* gene. Under low-iron conditions, Php4 is produced and becomes competent to associate with the CCAAT-binding core complex that is composed of Php2, Php3, and Php5. The Php4/CCAAT complex reprograms the cells for iron economy (Mercier *et al.* 2006). At the molecular level, Php4 is responsible for the transcriptional repression capability of the CCAAT complex (Mercier *et al.* 2008). The Php4/Php2/Php3/Php5 heteromeric complex coordinates the repression of 86 genes in cells that grow mitotically (Mercier *et al.* 2008). Among these, the majority encode proteins involved in iron-dependent metabolic pathways such as the tricarboxylic acid cycle (TCA), mitochondrial electron transport chain, heme biosynthesis, and iron-sulfur cluster assembly. Microarray analyses have also revealed that the *fep1^+^* gene is under the transcriptional control of Php4 being repressed in response to iron deficiency in a Php4-dependent manner (Mercier *et al.* 2008). Overall, Php4 and Fep1 mutually control each other’s expression at the transcriptional level in response to changes in iron levels.

Previous studies have used *S. pombe* mutants to distinguish between the effects of iron on Php4 protein and its transcriptional regulation by Fep1 (Mercier and Labbé 2009; Khan *et al.* 2014). This approach using cells proliferating in mitosis led to the discovery that Php4 exhibits a differential cellular localization as a function of iron availability. For instance, Php4 accumulates in the nucleus under low-iron conditions, whereas it transits from the nucleus to the cytoplasm in response to high levels of iron (Mercier and Labbé 2009). Although nuclear import of Php4 is independent of the other CCAAT-regulatory subunits Php2, Php3, and Php5, it is a cargo for the karyopherins Imp1, Cut15, and Sal3 (Khan *et al.* 2014). In mitotically growing cells undergoing a transition from low to high iron, Php4 transits from the nucleus to the cytoplasm in a process that is dependent on monothiol glutaredoxin Grx4 and exportin Crm1 (Mercier and Labbé 2009). When cells are exposed to iron-poor conditions, nuclear localization of Php4 is reestablished through a mechanism of import. A rationale for the Php4-mediated iron-sparing response is to prevent a futile expenditure of energy in producing iron-requiring proteins when iron is absent or present in insufficiently low concentrations. In the case of *php4*Δ mutant cells, iron-using genes are expressed continuously, rendering these cells hypersensitive to low-iron conditions (Mercier *et al.* 2008; Khan *et al.* 2014). This is presumably due to the lack of optimization of iron utilization when iron concentrations are insufficient to meet the metabolic needs of the cell.

Although it is known that deficiencies in iron-dependent proteins culminate in meiotic cell developmental defects and subfertility (Narisawa *et al.* 2002; Nordstrand *et al.* 2010; Kipp *et al.* 2011), iron homeostasis during meiotic differentiation has not been extensively studied. Here, we have combined the use of DNA microarray analysis and *S. pombe* azygotic and *pat1*-driven meiotic models to investigate whether iron deficiency and inactivation of Php4 would perturb the meiotic program. Results showed that iron is required for the normal progression of meiosis. Iron insufficient zygotes experienced a meiotic block at metaphase I. Similarly, zygotic cells carrying disrupted *php4*Δ*/php4*Δ alleles were arrested at metaphase I when iron was limited. The *php4^+^* gene was expressed at higher levels in iron-starved meiotic cells in comparison with cells treated with iron. Furthermore, *fep1*Δ*/fep1*Δ meiotic cells exhibited increased levels of *php4^+^* mRNA under low- and high-iron conditions. Microscopic analyses revealed that a functional GFP-Php4 protein colocalizes with chromosomes/chromatids in meiotic and sporulating cells under low-iron conditions. Using DNA microarrays, we identified a first set of genes whose transcription is expressed at higher levels in iron-replete meiotic cells. Second, we identified genes whose transcription is induced in a *php4*Δ*/php4*Δ mutant strain under low-iron conditions. These two combined data sets globally identified 137 genes that are regulated in an iron- and Php4-dependent manner, including 18 genes that are meiosis-specific. Experiments were designed to validate a direct role for Php4 in participating in the regulation of newly identified meiosis-specific target genes. Results of ChIP assays showed that the *hry1^+^* and *mug14^+^* promoters are directly bound by Php4 in response to iron starvation. Taken together, the results demonstrate that Php4 is required during the meiotic differentiation program to repress iron-using genes when iron concentrations are low, revealing a meiotic role for Php4 in the optimization of iron use under iron starvation conditions.

## Materials and Methods

### Yeast strains and growth conditions

The *S. pombe* strains used in this study are listed in Table 1. Standard yeast genetic methods were used for growth, mating, and sporulation of cells (Sabatinos and Forsburg 2010). Under nonselective conditions, strains were grown on yeast extract (YES) medium containing 0.5% yeast extract and 3% glucose that was further supplemented with 225 mg/L of adenine, histidine, uracil, leucine, and lysine. Strains for which plasmid integration was required were grown in synthetic Edinburgh minimal medium (EMM) lacking specific amino acids required for plasmid selection and maintenance. After mating, zygotic *h^+^/h*^-^ strains were returned to nonselective yeast extract medium before commitment to meiosis. Diploid cells underwent azygotic meiosis following a synchronized nitrogen-starvation shock in which EMM lacking nitrogen (EMM-N) was supplemented with 10 mg/L of adenine or 10 mg/L of adenine, histidine, leucine, uracil, and lysine. Diploid strains homozygous for the mating type (*h^+^/h^+^*) were generated by incubating haploid cell cultures with carbendazim (20 μg/ml) (Sigma-Aldrich) as described previously (Zhang *et al.* 2013).

**Table 1 S.** *pombe* strain genotypes

Strain	Genotype	Source or Reference
FY435	*h^+^ his7-366 leu1-32 ura4*-∆*18 ade6-M210*	Beaudoin *et al.* 2011
FY436	*h^-^ his7-366 leu1-32 ura4*-∆*18 ade6-M216*	Beaudoin *et al.* 2011
AMY15	*h^+^ his7-366 leu1-32 ura4*-∆*18 ade6-M210 php4*∆::*KAN^r^*	Mercier *et al.* 2006
ABY60	*h^-^ his7-366 leu1-32 ura4*-∆*18 ade6-M216 php4*∆::*KAN^r^*	This study
JB484	*h^+^ pat1-114 ade6-M210*	Bähler *et al.* 1991
JB485	*h^+^ pat1-114 ade6-M216*	Bähler *et al.* 1991
ABY61	*h^+^ pat1-114 ade6-M210 php4*Δ::*KAN^r^*	This study
ABY62	*h^+^ pat1-114 ade6-M216 php4*Δ::*KAN^r^*	This study
ABY63	*h^+^ pat1-114 ade6-M210 fep1*Δ::*KAN^r^*	This study
ABY64	*h^+^ pat1-114 ade6-M216 fep1*Δ::*KAN^r^*	This study
FY435/FY436	*h^+^/h^-^ his7-366/his7-366 leu1-32/leu1-32 ura4*-∆*18/ura4*-∆*18 ade6-M210/ade6-M216*	This study
php4∆/∆	*h^+^/h^-^ his7-366/his7-366 leu1-32/leu1-32 ura4*-∆*18/ura4*-∆*18 ade6-M210/ade6-M216 php4*Δ::*KAN^r^/php4*Δ::*KAN^r^*	This study

To synchronize *pat1-114*/*pat1-114* diploid cells (Bähler *et al.* 1991) for their entry into meiosis, liquid cultures were seeded to an *A_600_* of 0.2 and grown to midlog phase (*A_600_* of 0.5) in EMM supplemented with adenine (225 mg/L) at 25°. Cells were harvested, washed twice, and transferred to EMM minus nitrogen (EMM-N) that was supplemented with adenine (10 mg/L). At this point, cells were separated into two different lots that were pretreated with 2,2’-dipyridyl (Dip) (50 µM) and FeCl_3_ (Fe) (0.74 µM) for 16 hr at 25°, unless otherwise stated. After pretreatment of the cells, NH_4_Cl (0.5 g/L) was added to each lot and cells were further exposed to Dip (75 µM) and FeCl_3_ (100 µM), respectively. At this time, the temperature was shifted to 34° to induce meiosis as described previously (Beaudoin *et al.* 2011). Meiosis progression was monitored using the Hoechst 33342 stain (5 μg/ml) added at various times following meiotic induction.

### Plasmids

The *sad1^+^-mCherry* chimeric gene was isolated from pJK210*sad1^+^-mCherry* (Beaudoin *et al.* 2011) by PCR using primers that contained *Bam*HI and *Sst*I restriction sites at their ends. The purified DNA fragment was digested with *Bam*HI and *Sst*I and then cloned into the corresponding sites of pJK148 (Keeney and Boeke 1994). The resulting plasmid was denoted pJK148*sad1^+^-mCherry* and the fluorescent protein product served as a spindle pole body marker. Plasmid pJK-194*prom*php4^+^* harbors a 194 bp DNA segment of the *php4^+^* promoter (Mercier and Labbé 2009). The asterisk (in the plasmid name) indicates that the promoter contains multiple point mutations in the two iron-responsive GATA sequences (positions −188 to −183 and −165 to −160), rendering the promoter constitutively expressed irrespective of the iron status (Mercier and Labbé 2009). The wild-type *php4^+^* open reading frame was isolated by PCR from genomic DNA of the parental FY435 strain. The PCR product was digested with *Bam*HI and *Asp*718 and then cloned into the corresponding sites of pJK-194*prom*php4^+^*. The resulting plasmid was denoted pJK-194*prom*php4-php4^+^*.

The wild-type version of *php4^+^* promoter up to position −194 (from the start codon of the *php4^+^* gene) was isolated by PCR. After amplification, the purified DNA fragment (−194 to −1) was digested with *Sac*II and *Bam*HI and then was exchanged with the *Sac*II-*Bam*HI mutated php4* promoter region in plasmid pJK-194*prom*php4-GFP-php4^+^* (Mercier and Labbé 2009). The resulting plasmid was named pJK-194prom*php4-GFP-php4^+^*. A similar strategy was used to create the plasmid pJK-194prom*php4-TAP-php4^+^*, except that the *Sac*II-*Bam*HI PCR-amplified DNA segment containing the wild-type version of *php4^+^* promoter (−194 to −1) was exchanged with the mutated php4* promoter DNA fragment into the plasmid pJK-194*prom*php4-TAP-php4^+^* (Mercier and Labbé 2009). Plasmid pJK-194prom*php4-TAP-php4^+^* was used as a template to amplify a DNA fragment encompassing the *TAP-php4^+^* fusion gene and its promoter region up to −194. This PCR amplification was performed using primer pairs that incorporated unique 5′ and 3′ *Sac*II and *Apa*I restriction sites, respectively. The PCR product was purified, digested with *Sac*II and *Apa*I, and then cloned into the corresponding sites of pBP*ade6^+^* (Beaudoin *et al.* 2006). The resulting plasmid was denoted pBP-194prom*php4-TAP-php4^+^*.

### RNA analysis

Total RNA was extracted using a hot phenol method as described previously (Chen *et al.* 2003). Gene expression profiles were analyzed using RNase protection assays as described previously (Mercier *et al.* 2008). Plasmids pSK*php4^+^*, pSK*isa1^+^*, and pSK*act1^+^* (Mercier *et al.* 2006) were used to produce antisense RNA probes that served to determine *php4^+^*, *isa1^+^*, and *act1^+^* mRNA levels, respectively. Plasmid pSK*hry1^+^* was constructed by inserting a 196-bp *Bam*HI-*Eco*RI fragment from the *hry1^+^* gene into the same sites of pBluescript SK. The antisense RNA hybridizes to the region between positions +66 and +262 downstream of the initiator codon of *hry1^+^*. Plasmid pSK*mug14^+^* was generated by inserting a 193 bp fragment from the *mug14^+^* gene (corresponding to the coding region between positions +214 and +407 downstream of the A of the start codon of *mug14^+^*). ^32^P-labeled antisense RNA probes were produced from the above *Bam*HI-linearized plasmids and with the use of [α-^32^P]UTP and T7 RNA polymerase. *act1^+^* mRNA was probed as an internal control for normalization during quantification of RNase protection products.

### Microarray experiments

We adopted an experimental design that involved two nodes: *pat1-114/pat1-114 php4^+^/php4^+^* (WT) iron replete (+Fe) *vs.* *pat1-114/pat1-114 php4^+^/php4^+^* (WT) iron-starved (+Dip) and *pat1-114/pat1-114 php4∆/php4∆* iron-starved (+Dip) *vs.* *pat1-114/pat1-114 php4^+^/php4^+^* iron-starved (+Dip). Meiotic time courses were performed as three independent biological repeats. All of them were used in the microarray protocol for which the Alexa Fluor 555 and 647 dyes were swapped. A fourth independent biological repeat was used for quantification of mRNAs using RNase protection assays. Total RNA was isolated from cells that had undergone synchronous meiosis for 7 hr under the indicated iron status (replete or starved conditions). The preparation of cDNA libraries from samples of RNA was performed as described previously (Lyne *et al.* 2003). cDNAs were hybridized onto glass DNA microarrays (Agilent Technologies) containing 15,000 spots that were ∼60-mer probes. Together, these probes result in ∼2×3-times coverage for each *S. pombe* locus, representing all known and predicted protein-coding genes and some noncoding RNA genes. Microarrays were scanned using a GenePix 4000B laser scanner (Axon instruments). Data were analyzed using the GenePix pro software. Unreliable signals were filtered out and data were normalized using an R script as described previously (Marguerat *et al.* 2012). The script applies cut-off criteria to discard data from weak signals. Genes that did not yield reproducible results of biological repeats were eliminated. Furthermore, genes with 50% of their data points missing were also discarded. Data acquisition and processing were further analyzed using GeneSpring GX software (Agilent Technologies). Normalized signals were exported from GeneSpring into Excel software (Microsoft) and analyzed. To determine ratios of expression levels, gene values from the *php4^+^/php4^+^* (WT) (+Fe) and *php4*∆*/php4*∆ (+Dip) were divided by the corresponding value of *php4^+^/php4^+^* (WT) (+Dip), which was set as the reference sample. The expression ratios of biological repeat experiments were averaged. Genes were classified as *php4^+^*-dependent if their expression changed 2.0-fold more than the average of two repeats during iron repletion *vs.* iron starvation and if they were induced 2.0-fold more than the average of two repeats in the *php4*∆*/php4*∆ strain during iron starvation compared with the *php4^+^/php4^+^* (WT) strain under the same conditions. Gene annotations were retrieved from the PomBase website (Wood *et al.* 2012).

### Fluorescence microscopy

Assessment of GFP-Php4 localization during meiosis and sporulation was performed by using *h^+^ php4*∆ and *h^-^ php4*∆ haploid cells expressing a functional *GFP-php4^+^* allele and crossing the two strains to produce diploid zygotes. After mating, the diploid state of cells was stabilized by incubation in YES medium. Subsequently, diploid cells were precultured in the presence of Dip (50 µM) in EMM containing nitrogen supplemented with 75 mg/L of adenine, histidine, uracil, leucine, and lysine. The azygotic meiosis of diploid cells was synchronously induced by transferring the cells to nitrogen-poor EMM in the presence of Dip (75 µM) or FeCl_3_ (100 µM). After the cells had just entered meiosis, aliquots were withdrawn at various time points and stained with Hoechst 33342 (5 µg/ml) to assess progression of meiosis of individual cells. At the indicated meiotic phase, the cells were examined by fluorescence microscopy using a 1000 × magnification as described previously (Beaudoin *et al.* 2013). Fields of cells shown in this study correspond to a minimum of five independent experiments.

### ChIP assays

*h^+^/h^-^ php4*∆*/php4*∆ cells expressing untagged (from pJK148-194*prom*php4-php4^+^*) or TAP-tagged Php4 (from pJK148-194*prom*php4*-TAP-*php4^+^*) were induced to synchronously enter azygotic meiosis and then fixed (formaldehyde) after 7 hr. After formaldehyde cross-linking and neutralization with glycine, cell lysates were prepared by glass bead disruption in lysis buffer containing 100 mM HEPES-KOH pH 7.5, 1% Triton X-100, 0.1% Na-deoxycholate, 1 mM EDTA, 140 mM NaCl, 2 × cOmplete ULTRA Tablets (protease inhibitors, Roche), 1 mM phenylmethylsulfonyl fluoride (PMSF), 50 mM NaF, and 0.2 mM Na_3_VO_4_, as described previously (Larochelle *et al.* 2012). Samples were then sonicated using a Branson 450 sonicator to shear chromatin DNA into fragments of ∼500–1000 bp. Immunoprecipitation of TAP-Php4 bound to chromatin was performed using immunoglobin G (IgG)-Sepharose beads. Handling of beads, including washings and elution, reversed cross-linking, and DNA precipitation were performed as described previously (Adam *et al.* 2001; Jbel *et al.* 2009). Quantification of immunoprecipitated DNA was carried out by real-time PCR (qPCR) using different sets of primers that spanned *hry1^+^* and *mug14^+^* promoter regions. TAP-Php4 density at *hry1^+^* and *mug14^+^* promoters was calculated as the enrichment of specific genomic *hry1^+^* and *mug14^+^* promoter regions relative to an 18S ribosomal DNA coding region in which no functional CCAAT box was present. Primers were designated by the name of the gene promoter, followed by the position of their 5′ ends relative to that of the translational initiation codon: *hry1-412* (5′-GTCAATGGTGACGTAGAGAAAGA-3′), *hry1-323* (5′-AGGCCATTGACACGATGTT-3′), *mug14-692* (5′-GTTAGCTTCTATTTATGATGTCACTGTAA-3′), and *mug14-577* (5′-CTCTGGTTCTTCACGATCTTCTC-3′). Two primers derived from an 18S ribosomal DNA coding region were used as internal background controls: 18S-a (5′- CAGCTTGCGTTGAATACGTCCC-3′) and 18S-b (5′- AGCCAATCCAGAGGCCTCACTA-3′). Each qPCR was run in triplicate using Perfecta SYBR Green Fast mix (Quanta) on a LightCycler 96 Real-Time PCR instrument (Roche). All ChIP experiments were repeated at least three times using independent chromatin preparations.

### Protein extraction and analysis

*pat1-114/pat1-114 php4*∆*/php4*∆ cells expressing the *TAP-php4^+^/ TAP-php4^+^* allele were synchronized to initiate and proceed to meiosis. Every hour over a time period of 9 hr following meiotic induction, cells were fixed (formaldehyde) in the presence of Dip or FeCl_3_. For each time point, 15 min before cells were harvested, PMSF (1 mM) was added directly to the cultures. Cell lysates were prepared by glass bead disruption in the same lysis buffer as described for ChIP assays. TAP-Php4 was enriched using immunoglobin G (IgG)-Sepharose beads and equal amounts of each sample preparation were resuspended in sodium dodecyl sulfate loading buffer and proteins were resolved by electrophoresis on 8% sodium dodecyl sulfate-polyacrylamide gels. Proteins were electroblotted onto nitrocellulose membranes for 1 hr at 4°. Membranes were blocked by treatment with 5% powdered skimmed milk (Difco) in TBS (10 mM Tris-HCl, pH 7.4, 150 mM NaCl, and 1% bovine serum albumin) containing 0.1% Tween 20 (TBST). Following washings with TBST, membranes were incubated with primary antibodies diluted in 1% powdered skimmed milk in TBST for 16 hr at 4°. The following antibodies were used for immunodetection of TAP-Php4 and α-tubulin: polyclonal anti-mouse IgG antibody (ICN Biomedicals) and monoclonal anti-α-tubulin antibody (clone B-5-1-2; Sigma-Aldrich), respectively. After incubation, the membranes were washed and incubated with the appropriate horseradish peroxidase-conjugated secondary antibodies (Amersham Biosciences), developed with enhanced chemiluminescence (ECL) reagents (Amersham Biosciences), and visualized by chemiluminescence using an ImageQuant LAS 4000 instrument (GE Healthcare) equipped with a Fujifilm High Sensitivity F0.85 43 mm camera.

### Data availability

All data are included in the present article and in the Supplemental Material. Strains and plasmids used for this study are also available if requested.

## Results

### Iron deficiency leads to a meiotic block at metaphase I

**Figure 1:**
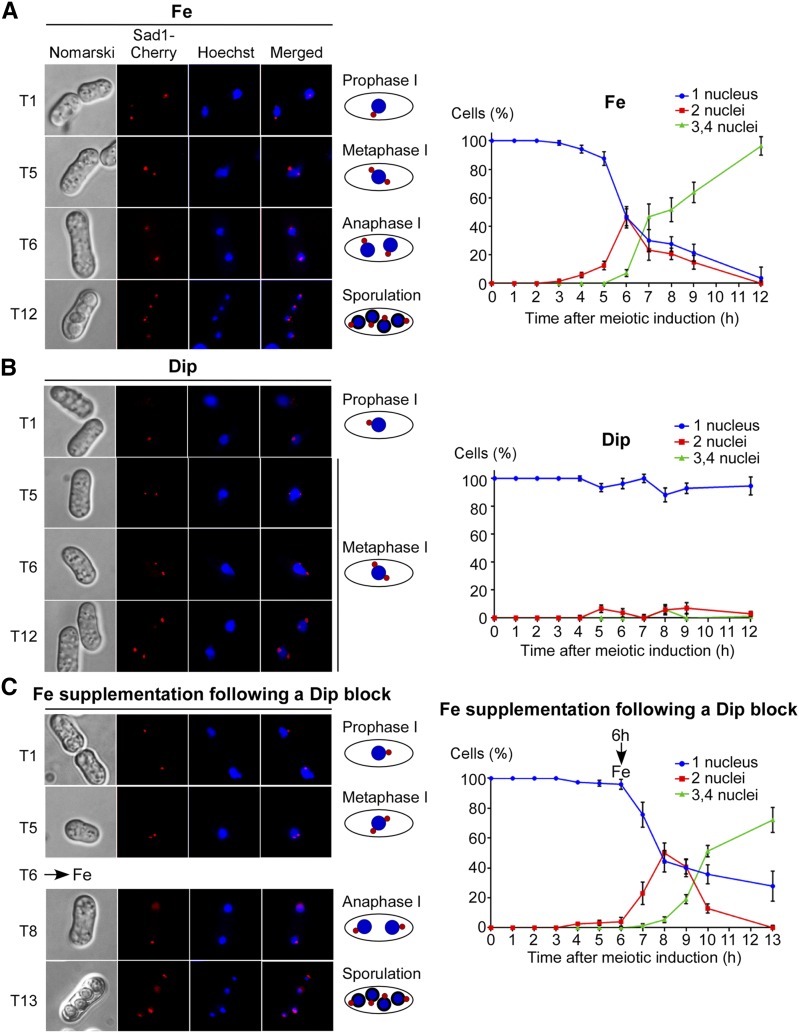
Iron insufficient zygotes undergo a meiotic arrest at metaphase I. Wild-type diploid cells expressing Sad1-Cherry were synchronously induced into azygotic meiosis. Shown are four representative stages of the meiotic program that occurred after 1, 5, 6, and 12 hr of meiotic induction. The spindle pole bodies’ marker Sad1-Cherry is in red (center left). The chromosomal material was probed by Hoechst 33342 staining (blue; center right). Cell morphology was examined by Nomarski optics (far left). Merged images of Hoechst dye and Sad1-Cherry are shown next to schematic representations of the meiotic steps on the far right. (A), Diploid cells underwent azygotic meiosis in the presence of Fe (100 µM). (B), In the case of iron insufficient zygotes, diploid cells were precultured in the presence of Dip (50 µM) for 16 hr and then transferred to media lacking nitrogen to initiate synchronous meiosis. Following the nitrogen-starvation shock, cells were treated with Dip (250 µM). (C) Aliquots of cells used in (B) (blocked at metaphase I) were incubated in the presence of exogenous Fe (300 µM), which resulted in release from metaphase I. The graphics (right) depict the meiotic profiles of cells after meiotic induction. Numbers of cells with 1, 2, or 3–4 nuclei were determined by counting Hoechst-stained nuclei after meiotic induction. At least 200 cells were counted every hour and under each above-mentioned condition. The reported values of cells are the means of three independent repeats ± SD. Dip, 2,2’-dipyridyl; Fe, iron(III) chloride; SD, standard deviation; T, time point (hours).

Although iron fulfills essential functions in eukaryotes, little is known about its role in meiosis. To investigate whether insufficient concentrations of iron would perturb the meiotic program, diploid cells were precultured in the presence of the iron chelator Dip (50 μM) or FeCl_3_ (0.74 µM) for 16 hr. At this point, Dip- and Fe-pretreated diploid cells underwent synchronous azygotic meiosis upon a nitrogen-starvation shock in the presence of Dip (250 μM) and FeCl_3_ (100 μM), respectively (Figure 1). Zygotes that were treated with Dip proceeded through the initial phases of the meiotic program until they reached metaphase I and then stopped their differentiation, exhibiting a meiotic arrest (Figure 1B). Over a time period of 5–12 hr after meiotic induction, the spindle pole body-associated protein Sad1 indicated that the chromosomal material failed to segregate (Figure 1B). To determine whether iron-insufficient zygotes could be relieved of metaphase I-like arrest by transfer into an iron-replete medium, zygotes that experienced a meiotic block were supplemented with FeCl_3_ (300 μM) after 6 hr of meiotic induction. Results showed that iron supplementation fostered rescue of the zygotes (Figure 1C). We noticed that a delay of ∼2 hr occurred when iron-insufficient zygotes were rescued by exogenous iron as compared to control zygotes for which iron was available during the meiotic program. Although a delayed rescue was observed, supplementation with iron restored the meiotic developmental program, including the two meiotic divisions and the generation of four haploid spores per ascus (Figure 1C). As positive controls, zygotes incubated in the presence of iron proceeded through meiosis and formed asci containing four spores after 10–12 hr of meiotic induction (Figure 1A). Percentages of cells with 1, 2, or 3–4 nuclei were quantitatively determined by counting Hoechst-stained nuclei (Figure 1). Taken together, the results showed that iron is required for normal progression of meiosis, based on the observation that the lack of iron leads to meiotic arrest at metaphase I.

### Meiotic cells harboring inactivated php4Δ/php4Δ alleles are arrested at metaphase I under low-iron conditions

**Figure 2:**
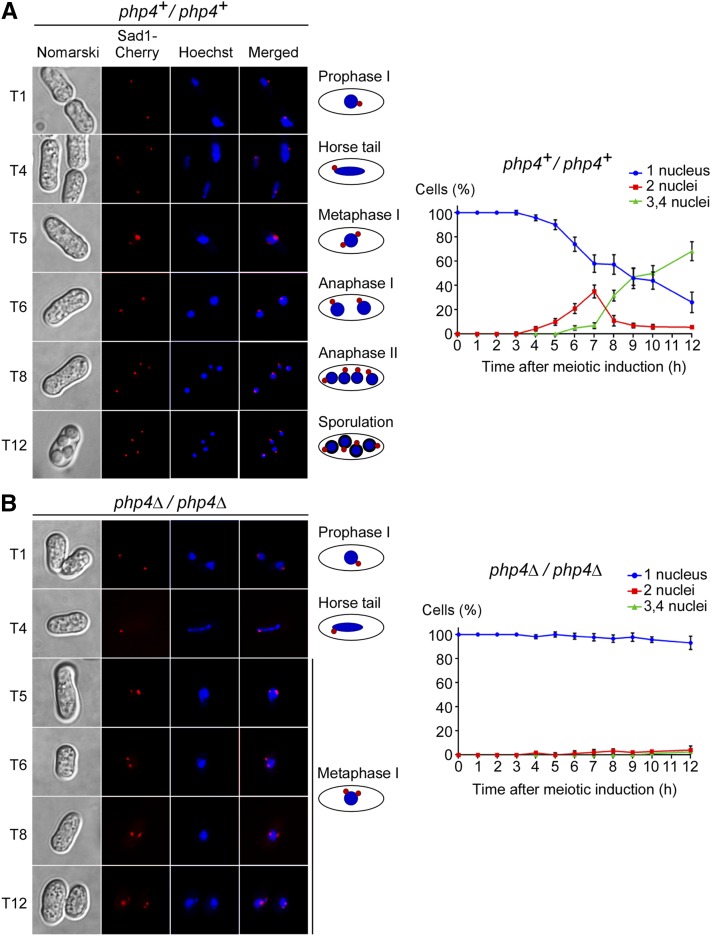
Php4 is required for progression of meiosis under low-iron conditions. *php4^+^/php4^+^* (A) and *php4*∆*/php4*∆ (B) cells expressing Sad1-Cherry (center left) were synchronously induced to undergo azygotic meiosis after a 16 hr pretreatment with Dip (50 µM). Immediately after entry into meiosis, both strains were incubated in the presence of additional iron chelator (Dip, 75 µM). For each strain, samples were taken at the indicated times after meiotic induction. Representative microscopic images revealed defective meiotic differentiation of *php4*∆*/php4*∆ cells compared to that seen with *php4^+^/php4^+^* cells. Hoechst staining was used to visualize chromosomal DNA (center right). Merged images of Sad1-Cherry and Hoechst dye are shown in the far right panels. Nomarski microscopy was used to examine cells or ascus morphology (far left). For both strains, graphics (right) represent percentages of cells with 1, 2, or 3–4 nuclei. For each time point (0–12 hr), at least 200 Hoechst-stained cells were counted. The reported values of cells represent the averages ± SD of triplicate experiments. Dip, 2,2’-dipyridyl; SD, standard deviation; T, time point (hours).

When iron levels are low, proliferating *S. pombe* cells that grow mitotically express the CCAAT-binding subunit Php4 (Mercier *et al.* 2006). Upon its biosynthesis, Php4 fosters repression of several genes encoding iron-using proteins as a means to minimize cellular iron consumption (Mercier *et al.* 2008). Taking into account the facts that Php4 is required for iron economy during mitosis and that iron plays an essential role during meiotic differentiation, we hypothesized that Php4 could also be important for normal progression of meiosis under conditions of iron starvation. To test this hypothesis, *php4*Δ*/php4*Δ diploid cells were used and results compared to *php4^+^/php4^+^* control cells. Diploid strains were precultured in the presence of Dip (50 μM) and were synchronously induced by transferring the strains at the same time to nitrogen-poor medium, thus allowing strains to undergo azygotic meiosis. Strains were treated with Dip (75 μM) following induction of meiosis. In the case of *php4^+^/php4^+^* cells, meiosis I occurred mainly between 5.5 and 7.5 hr, meiosis II between 7.5 and 9 hr, and spore formation after 10 hr of meiotic induction (Figure 2A). In the case of *php4*Δ*/php4*Δ mutant cells, the progression of meiosis stopped at metaphase I, although prophase I and horse tail steps were observed (Figure 2B). Fluorescence localization of Sad1-Cherry, a protein associated with the spindle pole body, revealed that the chromosomal material failed to segregate. A majority of *php4*Δ*/php4*Δ zygotes (>97%) underwent a meiotic block at metaphase I even after 12 hr of meiotic induction (Figure 2B). Percentages of cells with 1, 2, or 3–4 nuclei were quantitatively determined by counting Hoechst-stained nuclei. Taken together, the results showed that *php4*Δ*/php4*Δ mutant cells are unable to undergo meiotic differentiation under low-iron conditions, resulting in arrest at metaphase I.

### Temporal expression profile of Php4 during meiosis

**Figure 3:**
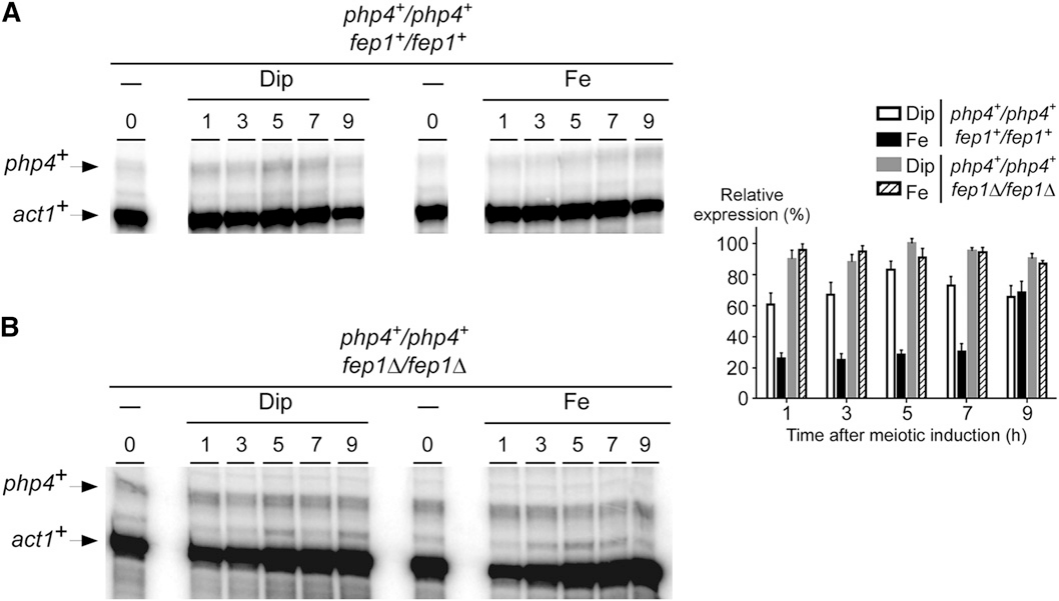
Fep1 plays a role in the repression of php4^+^ gene expression during meiosis under high concentrations of iron. Cultures of *pat1-114/pat1-114 php4^+^/php4^+^ fep1^+^/fep1^+^* (A) and *pat1-114/pat1-114 php4^+^/php4^+^ fep1*Δ*/fep1*Δ (B) cells were induced to initiate and undergo synchronous meiosis in the presence of Dip (75 µM) or Fe (100 µM). Total RNA was isolated from culture aliquots taken at the indicated time points. Following RNA isolation, *php4*^+^ and *act1*^+^ steady-state mRNA levels were analyzed by RNase protection assays. 0 hr: zero time point refers to onset of meiotic induction. Graphics (right) represent quantification of the results of three (n = 3) independent RNase protection assays, including experiments shown on the left side of the figure. The histogram values represent the averages ± SD. Dip, 2,2’-dipyridyl; Fe, iron(III) chloride; mRNA, messenger RNA; RNase, ribonuclease; SD, standard deviation.

To further investigate the meiotic function of Php4, we first assessed its transcription profile during meiosis as a function of time and iron availability. *pat1-114/pat1-114 php4^+^/php4^+^* diploid cells were synchronously induced into meiosis and treated with either Dip (75 μM) or FeCl_3_ (100 μM). Aliquots of cultures were taken after meiotic induction and the steady-state levels of *php4^+^* mRNA analyzed by RNase protection assays. Under low-iron conditions, results showed that steady-state levels of *php4^+^* transcripts were constitutively present between 1 and 9 hr after meiotic induction, exhibiting a small peak of expression at middle meiosis (*e.g.*, 5 hr time point) (Figure 3A). In the case of iron-starved cells, *php4^+^* transcript levels were expressed to a higher degree over time compared to transcript levels observed in iron-treated cells. Relative expression values were 42%, 51%, 66%, and 51% higher in the presence of Dip than iron after 1, 3, 5, and 7 hr of meiotic induction, respectively. The 9 hr time point represented an exception in which case *php4^+^* mRNA levels were expressed to a similar degree under iron-starved and iron-replete conditions (Figure 3A).

In cells proliferating in mitosis, *php4^+^* mRNA levels are repressed when cells are exposed to exogenous iron (Mercier *et al.* 2006). This iron-dependent repression of *php4^+^* gene expression is mediated by Fep1 (Mercier *et al.* 2006). To further examine whether *php4^+^* transcription was controlled by Fep1 during meiotic differentiation, a *pat1-114/pat1-114 fep1*Δ */fep1*Δ strain was incubated in the presence of Dip (75 μM) or FeCl_3_ (100 μM). In the presence of iron, inactivation of *fep1*Δ resulted in increased *php4^+^* mRNA levels that were similar to those seen in *fep1*Δ */fep1*Δ cells treated with Dip (Figure 3B). Under both conditions (Dip and FeCl_3_), disruption of *fep1* (*fep1*Δ */fep1*Δ) resulted in increased *php4^+^* transcript levels in comparison to those observed in the *pat1-114/pat1-114 fep1^+^/fep1^+^* control strain. Although Fep1 did not completely abolish *php4^+^* transcription in iron-replete meiotic cells, its inactivation resulted in increased *php4^+^* mRNA levels that were unresponsive to iron for repression.

**Figure 4:**
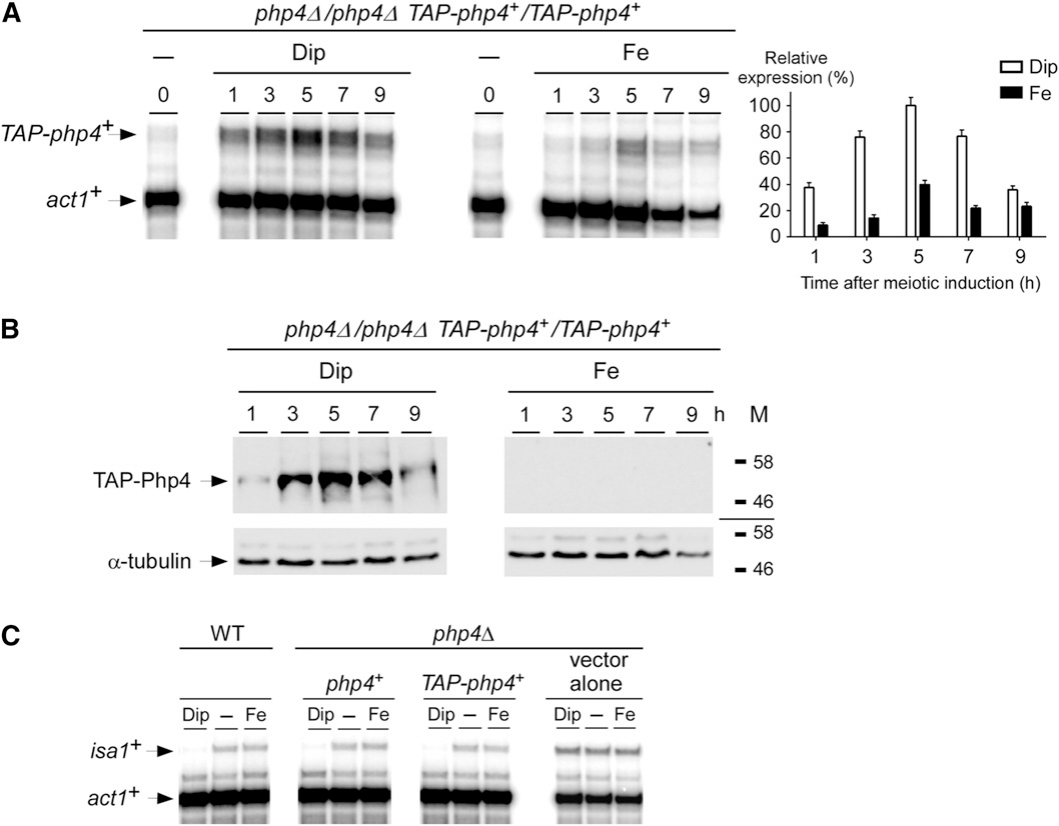
Assessment of the transcript and protein steady-state levels of a functional TAP-Php4 during meiosis. (A) Representative expression profile of *TAP-php4*^+^ mRNA in *pat1-114/pat1-114 php4*Δ*/php4*Δ *TAP-php4^+^/TAP-php4^+^* cells that were induced to undergo synchronous meiosis. Following induction of meiosis, cells were incubated in the presence of Dip (75 µM) or Fe (100 µM) and total RNA was isolated at the indicated time points. After RNA preparation, *TAP-php4*^+^ and *act1*^+^ steady-state mRNA levels were analyzed by RNase protection assays. 0 hr: zero time point refers to onset of meiotic induction. Graphics (right) represent quantification of results of three (n = 3) independent RNase protection assays, including experiments shown on the left side of the figure. The histogram values represent the averages ± SD. (B) Cell lysates from aliquots of the meiotic cultures expressing TAP-Php4 shown in (A) were analyzed by immunoblotting using anti-IgG and anti-α-tubulin antibodies. The positions of molecular weight standards are indicated on the right. (C) Mitotic WT and *php4*∆ strains were left untreated (−) or were incubated with either Dip (250 µM) or Fe (100 µM) for 90 min. *php4*∆ cells were transformed with integrative plasmids encoding *php4*^+^ and *TAP-php4*^+^ alleles or an empty integrative plasmid (vector alone). Total RNA prepared from midlogarithmic cells was assayed by RNase protection assays. Steady-state levels of *isa1*^+^ and *act1*^+^ mRNAs are indicated with arrows. Dip, 2,2’-dipyridyl; Fe, iron(III) chloride; IgG, immunoglobulin G; mRNA, messenger RNA; RNase, ribonuclease; SD, standard deviation; WT, wild-type.

To determine whether the steady-state protein levels of Php4 followed those of *php4^+^* mRNA, we used a *pat1-114/pat1-114 php4*Δ */php4*Δ strain in which a *TAP-php4^+^* fusion allele was returned into the genome by integration. In this strain, the expression profile of *TAP-php4^+^* mRNA was nearly identical to that of the *php4^+^* transcript in the wild-type (control) strain (Figure 3A and Figure 4A). Using the same culture conditions as for *php4^+^* or *TAP-php4^+^* mRNA analysis (Figure 3A and Figure 4A), results showed that TAP-Php4 protein levels were exclusively detected in iron-starved meiotic cells (Figure 4B). After cell entrance into meiosis (1 hr time point), the levels of Php4 protein were very low. Subsequently, a strong increase of Php4 protein levels was observed 3, 5, and 7 hr after meiotic induction. This was followed by a reduction of Php4 protein levels within 9 hr (Figure 4B). In contrast, under iron-replete conditions, the signal corresponding to TAP-Php4 was lost throughout the meiotic program, suggesting iron-mediated extinction of TAP-Php4 steady-state levels in response to high iron concentrations (Figure 4B).

To ensure that the in-frame TAP insertion did not interfere with Php4 function, the untagged (*php4^+^*) and tagged (*TAP-php4^+^*) coding sequences were separately integrated into *php4*Δ mutant cells. Integrants were analyzed for their ability to repress *isa1^+^* transcript levels in response to low concentrations of iron. Results showed that *php4*Δ cells expressing TAP-Php4 conferred iron starvation-dependent repression of *isa1^+^* expression in a manner similar to that of wild-type (untagged) Php4 protein (Figure 4C). In contrast, deletion of *php4^+^* (*php4*Δ) resulted in sustained expression of *isa1^+^* mRNA levels and a lack of response to iron starvation (Figure 4C). Taken together, these results revealed that TAP-Php4 is present in meiotic cells under low-iron conditions, whereas the protein steady-state levels are dramatically decreased in response to high concentrations of iron.

### Analysis of Php4 localization during meiosis under iron-limited and iron-replete conditions

**Figure 5:**
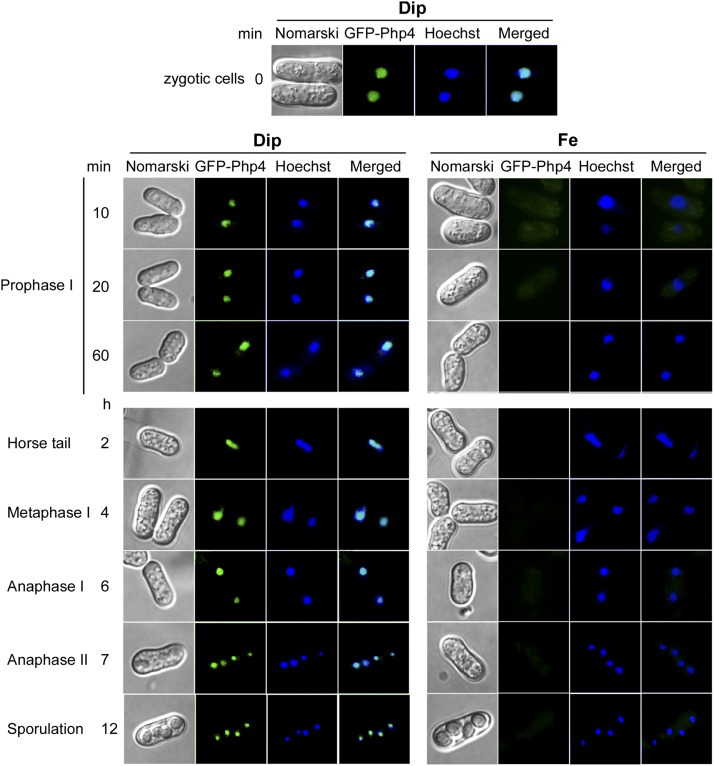
Analysis of GFP-Php4 localization during meiosis and sporulation as a function of iron availability. Diploid *php4*∆*/php4*∆ cells expressing GFP-Php4 (center left) were synchronously induced to undergo azygotic meiosis. Prior to meiotic induction, cells were pretreated with Dip (50 µM) for 16 hr (zygotic cells). Cells were then washed, divided in separate cultures and then incubated in the presence of Dip (75 µM) or Fe (100 µM) for the indicated time points. Hoechst staining was used to visualize DNA (center right). The merged images of GFP-Php4 and Hoechst dye are shown (far right). Cell morphology was examined by Nomarski optics (far left). Dip, 2,2’-dipyridyl; Fe, iron(III) chloride; GFP, green fluorescent protein.

We next determined the subcellular location of Php4 during meiosis and sporulation as a function of iron availability. As we previously showed, when GFP-Php4 is expressed in *php4*Δ mutant cells, the repression of *isa1^+^* mRNA occurs in response to iron starvation conditions in a manner identical to that observed in cells expressing the untagged (wild-type) version of Php4 (Mercier and Labbé 2009; Khan *et al.* 2014). These results demonstrated that GFP does not interfere with Php4 function. The fully functional *GFP-php4^+^* allele under the control of the *php4^+^* promoter was integrated in *h^+^ php4*∆ and *h^-^ php4*∆ cells, and localization of GFP-Php4 in zygotes and asci was determined. Diploid cells had undergone azygotic synchronous meiosis and they had been pretreated with Dip (50 µM) to trigger nuclear import of Php4. Results showed that GFP-Php4 was primarily detected in the nucleus of zygotic cells at the start of the observations (Figure 5). Once the cells were induced to undergo meiosis, one half of the cultures was further incubated with Dip (75 µM), whereas the other half was treated with FeCl_3_ (100 µM). Under conditions of iron starvation, GFP-Php4 colocalized with chromosomal material that was marked by Hoechst staining. This colocalization was observed through all different stages of meiosis, including prophase I, horse tail, metaphase I, and anaphase I and II (Figure 5). GFP-Php4 fluorescence in meiotic cells was observed as a single spot in each cell during prophase I and metaphase I in a manner similar to that observed for chromosomal material (Figure 5). Fluorescence associated with GFP-Php4 was seen as an elongated spot that appeared to correspond to the elongated nucleus during the “horse tail” stage (Figure 5). Following metaphase I, GFP-Php4 fluorescence was successively observed as a pair of spots per cell (anaphase I) and two pairs of spots per cell (anaphase II) (Figure 5). This result was interpreted to correspond to chromosomal material that had undergone two successive nuclear divisions, generating two homologous chromosomes and four sister chromatids, respectively. Cells displayed GFP-Php4 fluorescence as four distinct spots in the zygote during forespore membrane formation and sporulation (Figure 5). In response to high concentrations of iron, GFP-Php4 fluorescence had moved from the nucleus to the cytoplasm as can be predicted in the case of dividing cells that grow mitotically (Mercier and Labbé 2009). Strikingly, GFP-Php4 fluorescence disappeared in less than ∼20 min after the induction of meiosis (during prophase I) (Figure 5). This observation was consistent with the fact that TAP-Php4 steady-state levels were undetectable by immunoblot assays in iron-treated cells after 1, 3, 5, 7, and 9 hr of meiotic induction (Figure 4B). Taken together, these results added further support for the notion that Php4 is sensitive to iron and dramatically decreased under conditions of high levels of iron as compared with meiotic cells incubated under low-iron conditions.

### Effects of iron status and Php4 on S. pombe meiotic transcriptome

**Figure 6:**
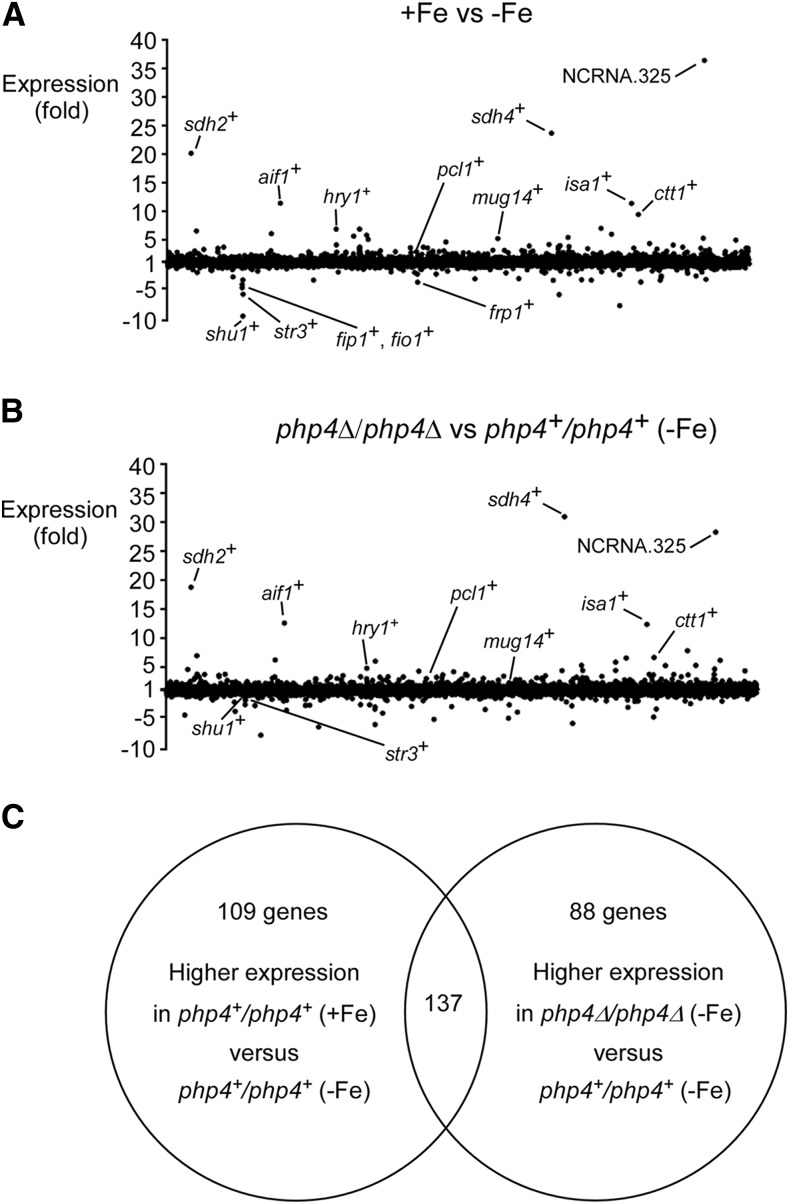
Transcriptomic response of *S. pombe* as a function of changes in iron levels and effect of *php4*∆*/php4*∆ disruption relative to wild-type cells during meiotic differentiation. (A) Cultures of *pat1-114/pat1-114 php4^+^/php4^+^* cells were precultivated in the presence of Fe (0.74 µM) and Dip (50 µM) for 16 hr. Pretreated cells were further exposed to Fe (100 µM) and Dip (75 µM), respectively, and then immediately induced to undergo synchronous meiosis. After 7 hr of meiotic induction, total RNA was extracted and used for microarray experiments. The graph represents a genome-wide picture of differentially expressed genes (X axis) in iron-replete *vs.* iron-limited cells. For simplicity, only a few differentially expressed transcripts are labeled on the graph. (B) Genome-wide picture of differentially expressed genes in *pat1-114/pat1-114 php4*Δ*/php4*Δ *vs.* *pat1-114/pat1-114 php4^+^/php4^+^* cells that had been precultured (50 µM) and cultured (75 µM) in the presence of Dip. Genome-wide transcripts (X axis) were analyzed by DNA microarrays. All differentially expressed genes are depicted, although only a few of them are labeled on the graph for the sake of clarity. (C) Venn diagram representing the number of genes that were induced in iron-treated *php4^+^/php4^+^* cells compared to *php4^+^/php4^+^* cells grown under iron-limiting conditions and the number of genes that exhibited higher expression levels in iron-starved *php4*∆/*php4*∆ compared to *php4^+^/php4*^+^ cells grown under the same conditions. Dip, 2,2’-dipyridyl; Fe, iron(III) chloride.

Given the fact that inactivation of *php4^+^* (*php4*Δ/*php4*Δ) altered the process of meiosis under iron-limiting conditions, we used a microarray approach to identify additional genes that were potentially under the control of Php4 and/or regulated as a function of iron availability during meiosis. The following conditions were used in the case of genes that are differentially regulated in response to changes in iron levels. Microarrays were hybridized with probes derived from RNA isolated from iron-replete *vs.* iron-starved *pat1-114/pat1-114* cells that had been synchronously induced to undergo meiosis. Differentially expressed genes were analyzed after 7 hr of meiotic induction. In this first set, 246 genes with high expression levels (averaging >2.0-fold) in the presence of iron were detected (Figure 6, A and C and Supplemental Material, Table S1). Among these genes, several of them encoded iron-using proteins that are involved in iron-dependent biochemical pathways, including the TCA cycle (*e.g.*, *sdh1^+^*/*2^+^*/*3^+^*/*4^+^*), mitochondrial respiration (*e.g.*, *cyc1^+^*, *qcr7^+^*, *cox5^+^*, and *cyt1^+^*), heme biosynthesis (*e.g.*, *hem3^+^* and *SPAP14E8.05c*), and iron-sulfur cluster assembly (*e.g.*, *isa1^+^*). Other genes encoded iron-containing proteins involved in diverse cellular functions such as biotin synthesis (*bio2^+^*), amino acid production (*leu2^+^*), and oxidative stress defense (*ctt1^+^*). With respect to meiosis, novel putative genes of unknown function were identified, including *hry1^+^*, *ppk24^+^*, and *mug30^+^* (Table S1). Under conditions of iron deficiency, we determined that 57 genes were expressed at high levels (averaging >2.0-fold). We consistently noticed that genes encoding proteins involved in reductive iron uptake were induced such as *frp1^+^*, *fip1^+^*, and *fio1^+^* (Figure 6 and Table S2). The *shu1^+^* gene encoding a cell-surface protein involved in iron acquisition from heme was also induced (Mourer *et al.* 2015). Furthermore, we observed significant changes in the transcriptional profiles of other genes, including *SPBPB2B2.06c* (a putative metal-dependent phosphatase), *frp2^+^* (a putative ferrireductase), and *ecl2^+^* (a putative metal-dependent extender of chronological lifespan) (Ohtsuka *et al.* 2015) (Figure 6 and Table S2).

In the case of genes that are potentially under the control of Php4, a second set of microarrays were hybridized with fluoro-cDNAs generated from mRNA preparations purified from iron-starved *pat1-114/pat1-114 php4*Δ*/php4*Δ mutant cells *vs.* iron-starved *pat1-114/pat1-114 php4^+^/php4^+^* cells. In this second set, 225 genes exhibited high levels of expression (averaging >2.0-fold) in the absence of Php4 (*php4*Δ*/php4*Δ) under low-iron conditions (Figure 6, B and C and Table S3). These potential Php4 target genes included genes already characterized, including *pcl1^+^*, *isa1^+^*, and *sdh4^+^* (Mercier *et al.* 2006), as well as several uncharacterized genes, especially those that are expressed exclusively during meiosis (24 meiotic genes were identified) (Table S3).

Overall, 137 genes were expressed at high levels in both sets of data, revealing that they shared a common trait. Transcription of these genes was iron- and Php4-dependent (Table 2). Consistently, the majority of these genes (119 of 137) had one or more copies of the CCAAT consensus sequence within their promoters (Table 2). Furthermore, several of these genes could be regrouped based on their predicted protein or RNA products or meiosis-specific profiles of expression. Interestingly, among genes derepressed by both iron repletion and *php4*Δ/*php4*Δ deletion, 18 of them were meiosis-specific (Table 2). Taken together, these results identified 246 genes that are expressed at high levels in iron-replete meiotic cells. Among them, 137 genes (including 18 meiosis-specific) exhibit decreased transcript abundance in iron-starved meiotic cells and are potentially under the control of Php4.

**Table 2:** Transcripts derepressed by both Fe-repletion and a *php4*Δ deletion

Gene ID	Gene Name	GeneDB Annotation	Fold Changes		Putative CCAAT Boxes
			WT (+Fe *vs.* −Fe)	*php4*Δ *vs.* WT (−Fe)	
Meiotically upregulated genes					
SPAC869.06c	*hry1^+^*	HHE domain cation binding protein (predicted)	6.826	4.734	386, 250*^a^*
SPBC359.06	*mug14^+^*	Adducin	5.127	2.229	649
SPCC1235.12c	*mug146^+^*	*Schizosaccharomyces* specific protein Mug46	3.715	4.370	854*^a^*, 804*^a^*, 753, 414*^a^*
SPBC6B1.03c		Pal1 family protein	2.680	2.636	709*^a^*, 539*^a^*
SPCC1281.04		Pyridoxal reductase (predicted)	2.658	3.033	102
SPAC3F10.05c	*mug113^+^*	T5orf172 family protein	2.505	2.309	792*^a^*, 597, 340, 251, 153
SPAPB1A10.08		*Schizosaccharomyces* specific protein	2.501	2.682	874*^a^*, 365*^a^*, 47*^a^*
SPBC21.07c	*ppk24^+^*	Serine/threonine protein kinase Ppk24	2.484	3.687	741*^a^*, 655*^a^*, 294
SPAC3F10.07c	*erf4^+^*	Palmitoyltransferase complex subunit Erf4	2.275	2.914	None
SPAC25A8.03c		DUF185 protein, mitochondrial	2.266	2.487	None
SPAC5D6.09c	*mug86^+^*	Acetate transmembrane transporter (predicted)	2.224	2.736	848*^a^*, 70, 32
SPCC320.07c	*mde7^+^*	RNA-binding protein Mde7	2.193	2.727	951, 818*^a^*, 264, 107
SPBP8B7.27	*mug30^+^*	HECT-type ubiquitin-protein ligase E3 (predicted)	2.166	2.433	226*^a^*, 134, 57
SPAC4F10.08	*mug126^+^*	*Schizosaccharomyces pombe* specific protein	2.157	2.617	158, 85
SPBC28E12.02		RNA-binding protein	2.129	2.936	970*^a^*, 754*^a^*, 271, 50
SPAC22F8.02c	*pvg5^+^*	PvGal biosynthesis protein Pvg5	2.094	2.205	665*^a^*, 267*^a^*, 9
SPCC1259.14c	*meu27^+^*	UPF0300 family protein 5	2.054	2.557	465*^a^*, 426*^a^*
SPBC19F8.06c	*meu22^+^*	Amino acid transmembrane transporter, predicted Meu22	2.004	2.641	826*^a^*, 724, 118
Noncoding RNAs					
SPNCRNA.325		Noncoding RNA (predicted)	36.510	28.320	541
SPNCRNA.31	prl31	Noncoding RNA, poly(A)-bearing (predicted)	5.192	4.596	965, 764, 448*^a^*, 274*^a^*
SPNCRNA.867		Intergenic RNA (predicted)	4.860	4.306	None
SPNCRNA.1314		Intergenic RNA (predicted), possible alternative UTR	3.993	7.783	719*^a^*
SPNCRNA.1457		Antisense RNA (predicted)	3.626	5.226	None
SPSNORNA.31	snoR39a	Small nucleolar RNA snR39	3.448	2.009	975*^a^*
SPNCRNA.242		Noncoding RNA (predicted)	3.429	2.017	738*^a^*
SPNCRNA.1063		Intergenic RNA (predicted), possible alternative UTR	3.309	2.821	542, 130*^a^*
SPNCRNA.495		Noncoding RNA (predicted)	3.184	6.122	937, 892*^a^*, 399*^a^*, 349*^a^*
SPNCRNA.1205		Intergenic RNA (predicted), possible alternative UTR	2.912	3.260	612, 567*^a^*, 74*^a^*, 24*^a^*
SPNCRNA.727		Intergenic RNA (predicted)	2.860	2.013	None
SPNCRNA.861		Intergenic RNA (predicted)	2.651	2.458	771, 504, 40
SPNCRNA.1325		Intergenic RNA (predicted), possible alternative UTR	2.456	3.597	634*^a^*
SPNCRNA.1157		Intergenic RNA (predicted)	2.425	2.310	922*^a^*
SPNCRNA.32	prl32	Noncoding RNA, poly(A)-bearing (predicted)	2.419	2.740	895*^a^*, 872*^a^*, 821*^a^*, 725, 675*^a^*, 273
SPSNORNA.16	snoR56	Small nucleolar RNA snR56 (predicted)	2.343	2.225	922*^a^*
SPNCRNA.30		Noncoding RNA (predicted)	2.313	3.648	546*^a^*, 387, 219, 179*^a^*
SPNCRNA.1087		Antisense RNA (predicted)	2.223	3.662	770*^a^*, 670, 590, 531*^a^*
SPNCRNA.940		Intergenic RNA (predicted)	2.173	2.142	515, 303, 76*^a^*
SPNCRNA.1617		Antisense RNA (predicted)	2.161	2.088	433*^a^*, 338, 265
SPNCRNA.1604		Intergenic RNA (predicted)	2.115	2.139	908, 901*^a^*
SPNCRNA.276		Noncoding RNA (predicted)	2.009	2.873	628*^a^*, 203
SPNCRNA.1343		Intergenic RNA (predicted)	2.005	2.108	384*^a^*
Electron transport chain/mitochondrial respiration					
SPCC191.07	*cyc1^+^*	Cytochrome c	6.953	5.821	907, 612, 525, 438, 380, 216*^a^*, 211*^a^*, 178
SPBC16H5.06	*rip1^+^*	Ubiquinol-cytochrome-c reductase complex subunit 5	3.480	3.197	906, 769*^a^*, 759, 657, 584, 453*^a^*, 313*^a^*
SPCC737.02c	*qcr7^+^*	Ubiquinol-cytochrome-c reductase complex subunit 6 (predicted)	2.622	2.715	729*^a^*, 611, 591, 462, 119*^a^*, 90
SPCC338.10c	*cox5^+^*	Cytochrome c oxidase subunit V (predicted)	2.511	2.422	947*^a^*, 696*^a^*, 562*^a^*, 112, 90
SPBC29A3.18	*cyt1^+^*	Cytochrome c1 Cyt1 (predicted)	2.509	2.195	813, 457, 435*^a^*, 145, 65
SPAC15A10.17	*coa2^+^*	Cytochrome C oxidase assembly factor Coa2 (predicted)	2.501	2.102	838, 146*^a^*, 139*^a^*, 132*^a^*
SPBC947.15c	*nde1^+^*	Mitochondrial NADH dehydrogenase (ubiquinone) Nde1 (predicted)	2.496	2.357	624, 489, 272*^a^*
SPBC16C6.08c	*qcr6^+^*	Ubiquinol-cytochrome-c reductase complex subunit 8, hinge protein (predicted)	2.372	2.100	158
SPAC20G8.04c	*cir2^+^*	Mitochondrial electron transfer flavoprotein-ubiquinone oxidoreductase Cir2 (predicted)	2.158	2.420	520*^a^*, 479*^a^*, 437*^a^*
Carbohydrates metabolic process					
SPBC32H8.^13^C	*mok12^+^*	α-1,3-glucan synthase Mok12	3.096	3.371	None
SPBC11C11.05		KRE9 family cell wall 1,6-β-glucan biosynthesis protein (predicted)	2.838	3.237	836
SPAC5H10.11	*gmh1^+^*	α-1,2-galactosyltransferase Gmh1 (predicted)	2.751	3.462	495
SPAC23H3.^11^C		Glucosidase (predicted)	2.449	2.194	902*^a^*, 654*^a^*, 642*^a^*
SPAC13F5.03c	*gld1^+^*	Mitochondrial glycerol dehydrogenase Gld1	2.252	4.537	None
SPAC1039.^11^C	*gto1^+^*	α-glucosidase (predicted)	2.249	2.387	706, 666*^a^*, 498*^a^*, 339
SPCC970.02		Mannan endo-1,6-α-mannosidase (predicted)	2.218	2.865	879*^a^*
SPBC19C7.12c	*omh1^+^*	α-1,2-mannosyltransferase Omh1	2.182	2.102	175*^a^*, 167*^a^*
Amino acid biosynthesis					
SPAC9E9.03	*leu2^+^*	3-isopropylmalate dehydratase Leu2 (predicted)	5.012	5.971	815*^a^*, 750*^a^*, 293
SPAPB1E7.07	*glt1^+^*	Glutamate synthase Glt1 (predicted)	2.982	3.254	913*^a^*, 881*^a^*, 266*^a^*, 199
SPAC17G8.06c		Dihydroxy-acid dehydratase (predicted)	2.781	3.534	327, 284*^a^*
SPBC21H7.07c	*his5^+^*	Imidazoleglycerol-phosphate dehydratase His5	2.636	2.623	994, 269, 165*^a^*
SPAC13G7.06	*met16^+^*	Phosphoadenosine phosphosulfate reductase	2.463	2.461	820*^a^*, 636*^a^*, 186, 63*^a^*
SPCC622.12c	*gdh1^+^*	NADP-specific glutamate dehydrogenase Gdh1 (predicted)	2.232	2.610	917*^a^*, 650, 528, 487*^a^*
SPCC1442.09	*trp3^+^*	Anthranilate synthase component I (predicted)	2.149	2.136	686, 138*^a^*
RNA-related proteins					
SPBC1718.03	*ker1^+^*	DNA-directed RNA polymerase I complex subunit Ker1	2.984	3.601	None
SPBC17D1.01		Transcriptional regulatory protein Spp41 (predicted)	2.870	2.079	None
SPAC3F10.06c	*rit1^+^*	Initiator methionine tRNA 2’-O-ribosyl phosphate transferase (predicted)	2.480	2.776	390*^a^*, 328*^a^*, 244*^a^*, 112
SPAC4G8.07c		tRNA (m5U54) methyltransferase Trm2 (predicted)	2.472	3.223	924*^a^*, 10*^a^*
SPCC757.09c	*rnc1^+^*	RNA-binding protein that suppresses calcineurin deletion Rnc1	2.376	2.130	23
SPCC11E10.06c	*elp4^+^*	Elongator complex subunit Elp4 (predicted)	2.091	2.046	614, 41, 19
SPCC320.^11^C	*nip7^+^*	RNA-binding protein involved in ribosome biogenesis Nip7 (predicted)	2.034	2.228	839, 608*^a^*
TCA cycle					
SPBP23A10.16	*sdh4^+^*	TIM22 inner membrane protein import complex anchor subunit Tim18	23.700	30.990	897*^a^*, 532, 77, 53
SPAC140.01	*sdh2^+^*	Succinate dehydrogenase (ubiquinone) iron-sulfur protein subunit (predicted)	20.160	18.790	100, 28
SPAC1556.02c	*sdh1^+^*	Succinate dehydrogenase Sdh1 (predicted)	6.471	6.920	178, 43
SPAC24C9.06c	*aco1^+^*	Aconitate hydratase Aco1 (predicted)	5.997	6.175	440, 135
SPCC330.12c	*sdh3^+^*	Succinate dehydrogenase (ubiquinone) cytochrome b subunit (predicted)	5.923	6.510	868*^a^*, 822*^a^*, 708*^a^*, 375, 253*^a^*, 175, 144, 11
SPBC3H7.03c		2-oxoglutarate dehydrogenase (lipoamide) (e1 component of oxoglutarate Dehydrogenase complex) (predicted)	2.674	2.764	None
Fe-S cluster biogenesis/Fe-S cluster-containing proteins					
SPAC26F1.14c	*aif1^+^*	Apoptosis-inducing factor homolog Aif1 (predicted)	11.380	12.590	951*^a^*, 355, 161
SPCC645.03c	*isa1^+^*	Mitochondrial iron-sulfur protein Isa1	11.350	12.330	762, 207
SPCC1235.02	*bio2^+^*	Biotin synthase	5.342	5.227	259
SPBC14F5.06	*rli1^+^*	Iron-sulfur ATPase involved in ribosome biogenesis and translation Rli1 (predicted)	3.537	4.235	980*^a^*, 393*^a^*, 255, 200*^a^*, 46*^a^*
mRNA metabolic process					
SPBC609.01		Ribonuclease II (RNB) family, involved in nuclear-transcribed mRNA Catabolic process (predicted)	2.899	3.048	646*^a^*, 424*^a^*, 386
SPAP8A3.05	*ski7^+^*	Ski complex interacting GTPase Ski7	2.481	2.641	586, 198, 126
SPBC16H5.10c	*prp43^+^*	ATP-dependent RNA helicase Prp43	2.129	2.144	678, 671*^a^*, 616
SPBC2F12.08c	*ceg1^+^*	mRNA guanylyltransferase Ceg1	2.008	2.186	None
Mitochondrial membrane-related					
SPBC3B9.19	*mge1^+^*	Mitochondrial GrpE domain chaperone protein (predicted)	3.076	2.745	971, 684, 415
SPCC1235.11	*mpc1^+^*	Mitochondrial pyruvate transmembrane transporter subunit Mpc1 (predicted)	2.938	3.139	434*^a^*, 273, 141
SPBC27B12.14		Mitochondrial membrane protein complex assembly protein (predicted)	2.122	3.004	810*^a^*
Oxidative stress response					
SPCC757.07c	*ctt1^+^*	Catalase	9.384	6.620	575*^a^*, 480
SPAC1486.01		Manganese superoxide dismutase	2.073	2.746	None
Sterol and fatty acid biosynthesis					
SPCC16A11.10c	*oca8^+^*	Cytochrome b5 (predicted)	3.357	3.327	None
SPAC1687.16c	*erg31^+^*	C-5 sterol desaturase Erg31	3.238	3.671	941*^a^*
Nitrogen assimilation					
SPCPB1C11.01	*amt1^+^*	Ammonium transmembrane transporter Amt1	4.256	5.213	964, 941, 301
SPAC23H4.06	*gln1^+^*	Glutamate-ammonia ligase Gln1	2.555	3.026	742, 690*^a^*, 680, 640
Heme biosynthesis					
SPAC24B11.13	*hem3^+^*	Hydroxymethylbilane synthase Hem3 (predicted)	2.988	3.212	None
SPAP14E8.05c		UPF0136 family mitochondrial protein, implicated in heme biosynthesis	2.571	3.208	977*^a^*, 501*^a^*
Other functions					
SPBPB21E7.02c		Phosphoglycerate mutase family	3.711	2.355	349, 153
SPAC869.08	*pcm2^+^*	Protein-L-isoaspartate O-methyltransferase Pcm2 (predicted)	3.633	2.488	447*^a^*, 108*^a^*
SPAC186.02c		Hydroxyacid dehydrogenase (predicted)	3.626	3.690	465
SPCC663.^13^C	*naa50^+^*	NatA N-acetyltransferase subunit Naa50 (predicted)	3.471	3.694	948*^a^*, 726*^a^*
SPCC663.14c	*trp663^+^*	TRP-like ion channel (predicted)	3.449	3.690	782*^a^*
SPAC17G8.08c		Human TMEM165 homolog, implicated in calcium transport	3.443	3.283	441*^a^*, 435*^a^*
SPAC1486.11	*fmc1^+^*	Mitochondrial matrix protein, F1F0 ATP synthase assembly factor Fmc1 (predicted)	2.959	3.052	312*^a^*, 174
SPBC1711.12		Serine-type peptidase activity	2.943	3.055	316
SPBC725.03		Pyridoxamine 5′-phosphate oxidase (predicted)	2.923	3.438	270
SPBC1652.02		APC amino acid transmembrane transporter (predicted)	2.814	2.296	929*^a^*, 671*^a^*, 323*^a^*, 128
SPBC1711.11		Autophagy associated protein (predicted)	2.657	2.450	536, 212, 39*^a^*, 26
SPCC1020.01c	*pma2^+^*	P-type proton ATPase, P3-type Pma2	2.654	3.201	119, 74, 42
SPAC869.02c		Nitric oxide dioxygenase (predicted)	2.612	2.938	315, 301*^a^*, 115*^a^*
SPAC1556.03	*azr1^+^*	Serine/threonine protein phosphatase Azr1	2.560	3.738	755*^a^*, 620*^a^*
SPBC1703.06	*pof10^+^*	F-box protein Pof10	2.520	2.113	814
SPBC1703.12	*ubp9^+^*	Ubiquitin C-terminal hydrolase Ubp9	2.520	2.093	612
SPCC830.08c	*yop1^+^*	ER membrane protein DP1/Yop1	2.398	2.355	None
SPBC19C7.09c	*uve1^+^*	Endonuclease Uve1	2.314	2.423	1000, 789, 242
SPBC1711.05		Nucleocytoplasmic transport chaperone Srp40 (predicted)	2.287	2.328	479, 356*^a^*, 68
SPAC8C9.03	*cgs1^+^*	cAMP-dependent protein kinase regulatory subunit Cgs1	2.234	2.154	667
SPBC26H8.02c	*sec9^+^*	SNAP-25 homolog, t-SNARE component Sec9	2.066	2.010	582, 461
SPBC1683.10c	*pcl1^+^*	Ferrous iron/manganese transmembrane transporter Pcl1	2.034	2.967	205
SPBC21D10.10	*bdc1^+^*	Bromodomain containing protein 1, Bdc1	2.005	2.317	None
Unknown functions					
SPAC750.05c		*Schizosaccharomyces pombe* specific 5Tm protein family	5.570	3.266	810*^a^*, 453, 444, 23*^a^*
SPBP19A11.02c		*Schizosaccharomyces pombe* specific protein, predicted GPI anchored	4.088	4.346	82*^a^*
SPBPB2B2.19c		*Schizosaccharomyces pombe* specific 5Tm protein family	3.910	2.918	809*^a^*, 453, 444, 23*^a^*
SPBC685.03		*Schizosaccharomyces* specific protein	3.839	3.018	590, 246*^a^*, 200*^a^*
SPBC18E5.07		DUF3210 family protein	2.997	4.136	850*^a^*, 228*^a^*
SPAC11D3.13	*hsp3104^+^*	ThiJ domain protein	2.715	2.307	658*^a^*
SPAC6C3.02c		Mitochondrial CHCH domain protein (predicted)	2.692	2.250	728, 256
SPCC191.06		*Schizosaccharomyces pombe* specific protein	2.670	4.362	671, 498*^a^*, 311, 16
SPCC736.05	*wtf7^+^*	Wtf element Wtf7	2.581	3.383	None
SPCC4G3.03		WD40/YVTN repeat-like protein	2.505	2.426	458, 441
SPBC1A4.04		*Schizosaccharomyces* specific protein	2.492	2.929	920*^a^*, 412, 358, 203*^a^*
SPAC15A10.07		*Schizosaccharomyces* specific protein	2.474	3.266	973, 694*^a^*, 383, 29
SPBC31A8.02		Pseudogene	2.432	2.690	None
SPCC1322.10		Cell wall protein Pwp1	2.208	2.411	934*^a^*, 461*^a^*
SPCC417.15		Dubious	2.190	2.763	987*^a^*, 964*^a^*, 913*^a^*, 817, 767*^a^*, 365
SPAPB1E7.^11^C		*Schizosaccharomyces* specific protein	2.017	2.349	811*^a^*, 325*^a^*
SPAC694.04c		Conserved eukaryotic protein	2.010	2.336	301

ID, identified; DB, database; WT, wild type; Fe, iron; S, sulfur; PvGal, pyruvic acid 4,6-ketal-linked galactose; UTR, untranslated region; NADH, nicotinamide adenine dinucleotide hydride; NADP, nicotinamide adenine dinucleotide phosphate; tRNA, transfer RNA; ATPase, adenosine triphosphatase; mRNA, messenger RNA; GTPase, guanosine triphosphatase; ATP, a denosine triphosphate; APC, amino acid-polyamine-organocation; ER, endoplasmic reticulum; cAMP, cyclic adenosine monophosphate; SNAP-25, synaptosome-associated protein 25 kDa; t-SNARE, target membrane soluble N-ethylmaleimide-sensitive attachment protein receptor.

a CCAAT boxe(s) in reverse orientation relative to the initiator codon of the indicated gene.

### Iron deficiency affects expression profiles of hry1^+^ and mug14^+^ meiosis-specific transcripts in a Php4-dependent manner

**Figure 7:**
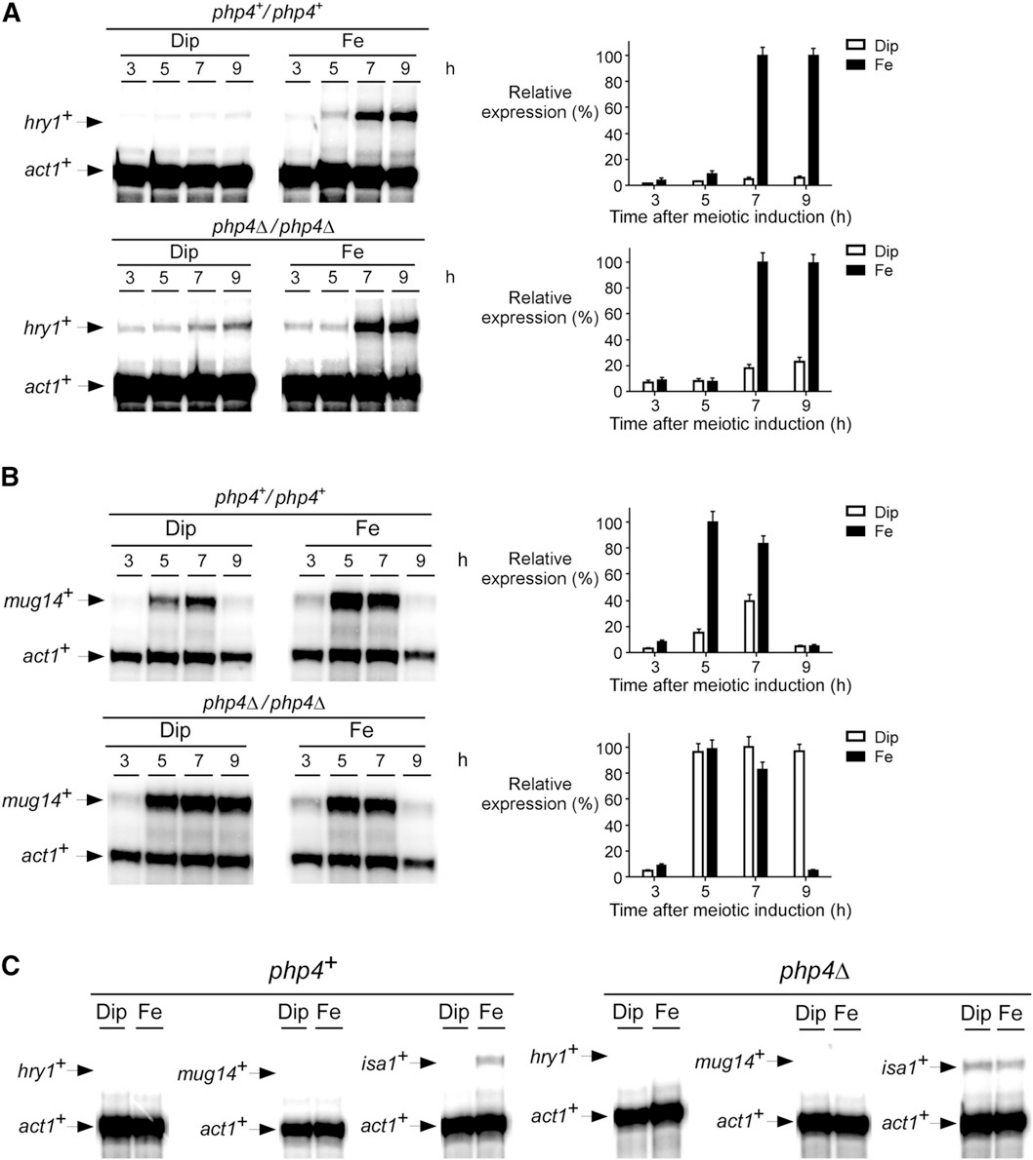
Effect of php4Δ disruption on the expression of two genes encoding meiosis-specific proteins. *pat1-114/pat1-114 php4^+^/php4^+^* and *pat1-114/pat1-114 php4*∆/*php4*∆ cells underwent synchronous meiosis under iron depleted (Dip, 75 µM) or iron-replete (Fe, 100 µM) conditions. At the indicated time points, *hry1^+^* (A), *mug14^+^* (B), and *act1^+^* mRNA levels were analyzed by RNase protection assays. Graphics (right) represent quantification of three (n = 3) independent RNase protection assays, including experiments shown on the left side of panels A and B. Histogram values are shown as averages ± SD. (C) Wild-type (*php4^+^*) and *php4*∆ cells proliferating in mitosis were incubated in the presence of Dip (250 µM) or Fe (100 µM) for 90 min. Shown are representative RNase protection assays of *hry1^+^*, *mug14^+^*, and *act1^+^* mRNA steady-state levels during mitosis. *isa1^+^* gene expression was probed as a control of gene known to be repressed under conditions of iron starvation in a Php4-dependent manner. Dip, 2,2’-dipyridyl; Fe, iron(III) chloride; RNase, ribonuclease; SD, standard deviation.

The microarray data suggested that 18 genes encoding meiosis-specific proteins were differentially expressed in relationship with the presence of Php4 under low-iron conditions (Table 2). To confirm that the results of the microarrays identified Php4-regulated genes, we performed RNase protection assays (using an independent biological repeat) to assess the relative expression of two meiosis-specific genes, *hry1^+^* (*SPAC869.06c*) and *mug14^+^*. Although both genes encode proteins of unknown function, the sequence of Hry1 contains a putative hemerythrin domain that may directly bind iron, suggesting that Hry1 may function as an iron-using protein. *pat1-114 php4^+^/php4^+^* and *pat1-114 php4*Δ*/php4*Δ diploid cells were synchronously induced to undergo meiosis under iron-starved and iron-replete conditions. At different time points after meiotic induction, steady-state levels of *hry1^+^* mRNA were analyzed. Results showed that *hry1^+^* mRNA levels were primarily detected in *php4^+^/php4^+^* cells treated with iron after 7 and 9 hr of meiotic induction. At these time points, levels of *hry1^+^* mRNA increased 19.2- and 16.4-fold, respectively, as compared to *hry1^+^* mRNA levels observed in iron-starved *php4^+^/php4^+^* cells (Figure 7A). Under iron starvation conditions, inactivation of *php4∆/php4∆* triggered an increase of *hry1^+^* expression after 3, 5, 7, and 9 hr of meiotic induction (4.6-, 2.6-, 3.5-, 3.8-fold, respectively) as compared to *hry1^+^* mRNA levels in *php4^+^/php4^+^* cells that had been exposed to identical conditions (75 μM Dip). This observation showed that Php4 was required for maximal repression of *hry1^+^* in response to iron starvation. Total RNA isolated from mitotically growing cells revealed that *hry1^+^* mRNA was undetectable regardless of cellular iron and Php4 status (Figure 7C). These observations were expected in view of the function of a gene predicted to be expressed exclusively during meiosis.

The meiotic expression profile of *mug14^+^* was first analyzed in *pat1-114 php4^+^/php4^+^* cells incubated in the presence of iron (FeCl_3_, 100 μM). Under these conditions, results showed that *mug14^+^* mRNA levels were markedly elevated after 5 and 7 hr of meiotic induction (Figure 7B). When *pat1-114 php4^+^/php4^+^* cells were synchronized through meiosis but under low levels of iron (75 μM Dip), *mug14^+^* mRNA levels were mainly detected after 5 and 7 hr of meiotic induction but to a lesser extent (6.4- and 2.1-fold less, respectively) in comparison with transcript levels observed in iron-replete cells (Figure 7B). When a *pat1-114 php4*Δ*/php4*Δ mutant strain was examined under iron-replete conditions, *mug14^+^* transcript levels were primarily detected at the 5 and 7 hr meiotic time points as observed in the case of iron-treated *pat1-114 php4^+^/php4^+^* cells (Figure 7B). However, under iron starvation conditions, disruption of *php4*Δ*/php4*Δ resulted in induced *mug14^+^* mRNA levels after 5, 7, and 9 hr of meiotic induction (6.1-, 2.5-, and 20.0-fold, respectively) compared to those recorded in the case of *php4^+^/php4^+^* cells incubated under the same conditions (Figure 7B). As observed in the case of the *hry1^+^* gene, expression of *mug14^+^* was detected exclusively during meiosis and was not seen in cells proliferating in mitosis (Figure 7C). Taken together, these results indicated that the repression of meiotic *hry1^+^* and *mug14^+^* genes occurs to a certain degree through the activity of the CCAAT-binding factor Php4, which represses transcription from these loci in response to iron starvation.

### Php4 interacts with the hry1^+^ and mug14^+^ promoters in vivo in an iron-dependent manner

**Figure 8:**
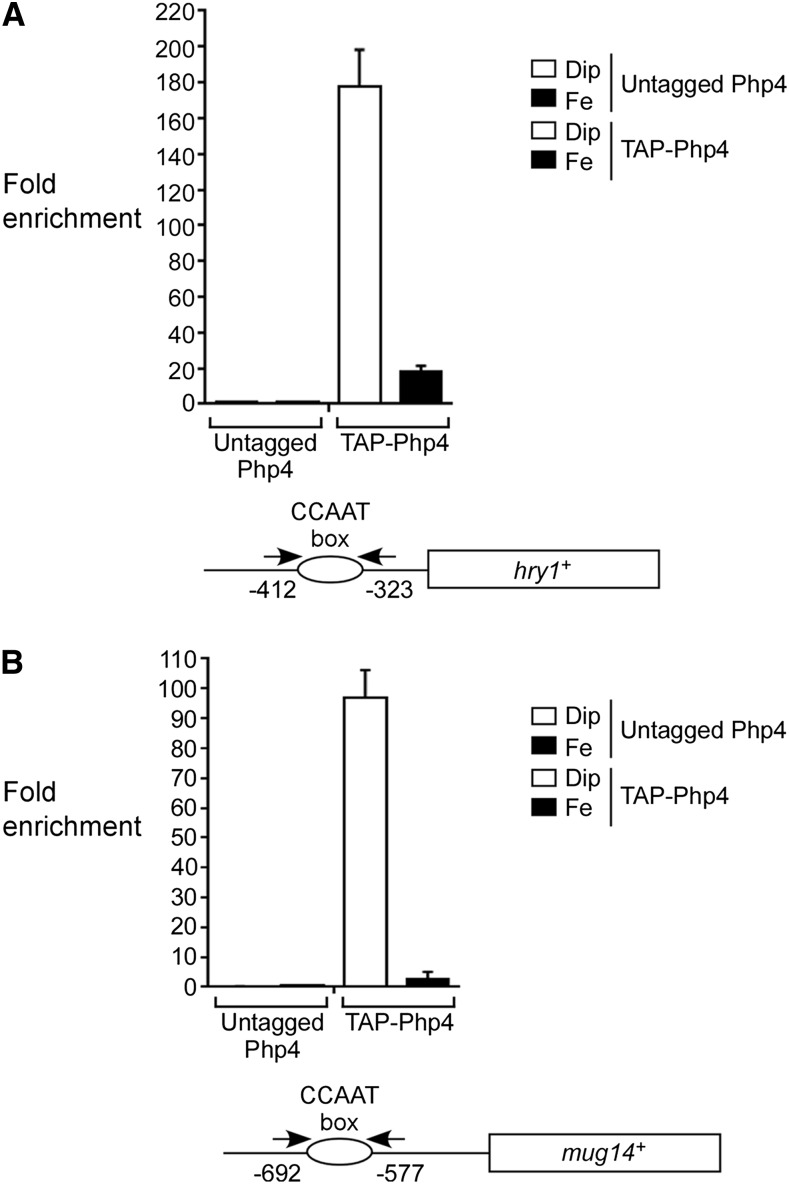
Php4 is recruited to the promoter of *hry1^+^* and *mug14^+^* genes under low-iron conditions. *pat1-114/pat1-114 php4*∆*/php4*∆ cells expressing an integrated untagged or a TAP-tagged *php4^+^* allele were synchronously induced to undergo meiosis. Cells were incubated in the presence of Dip (75 µM) or Fe (100 µM). After 7 hr of meiotic induction, chromatin was immunoprecipitated using Sepharose-bound anti-mouse IgG antibodies. Specific regions of *hry1^+^* (A) and *mug14^+^* (B) promoters were analyzed by qPCR to determine TAP-Php4 occupancy. Association of TAP-Php4 to promoters was calculated as the enrichment of specific *hry1^+^* and *mug14^+^* promoter regions relative to a 18S ribosomal DNA coding region. ChIP data were calculated as values of the largest amount of chromatin measured (fold enrichment). Results are shown as the averages ± SD of a minimum of three independent experiments. Diagram representations of *hry1^+^* and *mei4^+^* promoters (below histograms) indicate locations of the primers that were used for qPCR analysis. Nucleotide numbers refer to the position relative to the A of the initiator codon of each gene. ChIP, chromatin immunoprecipitation; Dip, 2,2’-dipyridyl; Fe, iron(III) chloride; IgG, immunoglobulin G; qPCR, quantitative polymerase chain reaction; SD, standard deviation.

In previous studies, we had developed a biological system in which *php4^+^* and *TAP-php4^+^* alleles were expressed under the control of a GATA-less *php4^+^* promoter (Mercier and Labbé 2009). We showed that a *php4*Δ mutant strain expressing *php4^+^* or a functional *TAP-php4^+^* allele was disengaged from transcriptional regulation by Fep1, therefore ensuring its constitutive expression irrespective of the cellular iron status. We took advantage of this system to test whether TAP-Php4 could be detected at the *hry1^+^* and *mug14^+^* promoters *in vivo* using a ChIP approach. In the case of the *S. pombe* CCAAT-binding complex, its capacity to associate with chromatin is conferred by the Php2/Php3/Php5 subunits that are required for the formation of a DNA binding complex at the CCAAT box promoter element (McNabb *et al.* 1997; Mercier *et al.* 2006). In response to iron starvation, Php4 associates with the Php2/Php3/Php5 heteromeric complex (Mercier *et al.* 2006). In contrast, when cells undergo a transition from low to high iron, Php4 is regulated at the posttranslational level via a multistep mechanism resulting in its inactivation (Mercier and Labbé 2009). *php4*Δ*/ php4*Δ diploid cells expressing either an untagged or a TAP-tagged version of Php4 under the control of a GATA-less *php4^+^* promoter were synchronized to initiate and proceed through azygotic meiosis under iron-deficient or iron-replete conditions. After 7 hr of meiotic induction, results showed that TAP-Php4 occupied the *hry1^+^* and *mug14^+^* promoters at high levels in response to iron starvation (Figure 8, A and B, respectively). The association of TAP-Php4 with *hry1^+^* and *mug14^+^* promoters exhibited 177- and 97-fold enrichment, respectively, relative to a 18S ribosomal DNA coding region that does not contain any CCAAT element (used as a negative control) (Figure 8). Promoter occupancy by TAP-Php4 was detected using primers amplifying DNA regions located between positions −412 and −323 (*hry1^+^*) and positions −692 and −577 (*mug14^+^*) relative to the initiator codons of *hry1^+^* and *mug14^+^*, respectively. These two amplified promoter regions were predicted to contain a putative functional CCAAT element (Figure 8). When meiotic cells were incubated in the presence of iron, TAP-Php4 chromatin occupancy of *hry1^+^* and *mug14^+^* promoters decreased drastically, exhibiting 17.7- and 2.6-fold TAP-Php4 enrichment, respectively, relative to a 18S ribosomal DNA coding sequence. These levels of enrichment were 10- and 37-fold weaker, respectively, compared to those of cells incubated under low-iron conditions. Results showed that untagged Php4 immunoprecipitated only background levels of *hry1^+^* and *mug14^+^* promoter regions (Figure 8). Taken together, these results showed that Php4 is recruited to the *hry1^+^* and *mug14^+^* promoters primarily in response to low concentrations of iron. Furthermore, the results further validated the microarray data that has revealed the existence of novel meiosis-specific Php4 target genes.

## Discussion

Meiosis is a critical biological process whereby genetic information is transmitted to the next generation in sexually reproducing species. In mice, zinc ions acquired during the early stage of meiosis are critical for egg development. In the case of severe zinc deficiency, oocytes undergo a meiotic arrest at telophase I, preventing the second meiotic division (MII) (Kim *et al.* 2010). In *S. pombe*, studies have shown that copper-insufficient zygotic cells experience a meiotic block at metaphase I (Beaudoin *et al.* 2011). Here, we report a similar result for *S. pombe* zygotic cells that were synchronously induced into meiosis under severe conditions of iron starvation. Iron insufficiency led to an arrest at metaphase I. The observation that iron can be a limiting factor for normal progression of meiosis suggests that its homeostatic status may be under the control of a regulatory mechanism that prevents futile expression of iron-using proteins in response to iron deficiency.

In *S. pombe* and several filamentous yeasts, mechanisms of iron-sparing include downregulation of iron-using proteins by transcript repression through a specialized subunit of the CCAAT-binding factor, which is synthesized under low-iron conditions (Mercier *et al.* 2006, 2008; Hortschansky *et al.* 2007; Jung *et al.* 2010; Schrettl *et al.* 2010; Brault *et al.* 2015). This subunit, called Php4 (*S. pombe*) or HapX (*Aspergillus* species and *Cryptococcus neoformans*), binds to a heterotrimeric DNA-binding complex, which becomes competent to repress target gene expression. In *A. fumigatus*, inactivation of HapX (*hapX*Δ) decreases asexual reproduction under iron starvation conditions (Schrettl *et al.* 2010). The production of conidia is significantly reduced, exhibiting 62% less formation compared to wild-type cells (Schrettl *et al.* 2010). Although the step where conidiogenesis is blocked remains unclear, the absence of HapX results in attenuation of *A. fumigatus* to produce conidia, making this fungus less effective to disperse conidia into new environments such as a host organism. In the case of Php4, its disruption in meiotic cells led to an arrest at metaphase I under low-iron conditions. This meiotic block may be due to lack of optimization of iron utilization when iron is limited. To confirm that the absence of Php4 resulted in a constitutive expression of iron-using genes during meiosis, DNA microarray experiments were performed. Transcripts corresponding to 225 genes were up-regulated (>twofold) in the absence of *php4* (Table S3). Out of these 225 genes, 21% were predicted to encode proteins involved in iron-dependent biochemical pathways. The number of 225 genes was higher than the 56 genes (>twofold) and 132 genes (>1.5-fold) previously identified in iron-starved cells proliferating in mitosis (Mercier *et al.* 2008). The higher number of identified Php4 target genes may be due to the experimental approaches used here as opposed to those of previous microarray results (Rustici *et al.* 2007; Mercier *et al.* 2008). First, the microarray gasket slide from Agilent Technologies was improved in that it contained a larger number of probes (15,000) which allowed a ∼2–3 × increased coverage for each *S. pombe* ORF. Second, all *S. pombe* ORFs were represented, including multiple sequence orphan genes and several small *S. pombe* specific ORFs that were not known in previous genome-wide microarray screens. Third, a large number of meiosis-specific genes identified in the present study could not be detected in previous screens due to the fact that they were not expressed in dividing cells that grew mitotically. In a second set of experiments, we identified 246 genes that were expressed at high levels under iron-replete conditions in *php4^+^/php4^+^* cells (Table S1). Based on the hypothesis that Php4 target genes would be expressed at higher levels in iron-replete *php4^+^/php4^+^* cells than in iron-starved *php4^+^/php4^+^* cells, and that they would be expressed at higher levels in iron-deficient *php4*Δ*/php4*Δ cells than in iron-deficient *php4^+^/php4^+^* cells, the overlap of the two sets of arrays included 137 genes (Table 2). Among these 137 genes loci, 23 genes corresponded to noncoding RNAs, whereas 113 genes were predicted or known to encode proteins. In the group of gene-encoded proteins, 35% of these had been assigned a known or probable function in iron-related processes. We also noted that 18 genes encoded meiosis-specific proteins (Table 2). Microarray results showed that the meiotic *hry1^+^* gene was the most highly expressed (6.8-fold) of all of the meiotic mRNAs detected under iron-replete conditions after 7 hr of meiotic induction. A relationship between Php4 and expression of *hry1^+^* was observed when *php4^+^* was deleted (*php4*Δ). This observation revealed that *hry1^+^* was subjected to Php4-dependent repression under low-iron conditions. Interestingly, *hry1^+^* encodes a protein that is predicted to possess a hemerythrin-like (Hr) domain (Stenkamp 1994; Xiong *et al.* 2000; French *et al.* 2008). Hr domains contain a di-iron center that often reversibly binds oxygen (Xiong *et al.* 2000). Proteins that contain Hr domains were first identified in some marine invertebrates (Stenkamp 1994). Subsequently, Hr domain-containing proteins have been found in bacteria, animals, and plants (French *et al.* 2008; Salahudeen *et al.* 2009; Kobayashi *et al.* 2013). Potential functions of Hr domains include the detection/transport of oxygen and the detoxification, storage, and sensing of iron (French *et al.* 2008). In humans, a Hr-like domain has been uncovered in the FBXL5 protein (Thompson *et al.* 2012; Ruiz and Bruick 2014). Elegant studies have demonstrated that the FBXL5 hemerythrin domain acts as an iron sensor and fosters degradation of iron regulatory protein 2 under iron-replete conditions through the ubiquitin-proteasome system (Salahudeen *et al.* 2009). Interestingly, Hry1 represents the first example of a hemerythrin-like protein in yeast. Since this putative iron-using protein may participate in regulating ion homeostasis during meiosis, it represents an attractive candidate for future study. We found that *mug14^+^* was a second meiotic gene that exhibited Php4-dependent changes at the transcriptional level. This gene encodes a methylthioribulose-1-phosphatase dehydratase-like protein that is the third enzyme involved in the methionine salvage pathway present in numerous organisms (Pirkov *et al.* 2008; Albers 2009; Mary *et al.* 2012). This pathway requires iron and involves six enzymes, including an iron-requiring acireductone dioxygenase (Adi1 in yeast), which performs the fifth step of the pathway. In response to iron starvation, meiotic *S. pombe* represses Mug14 expression and that may trigger arrest (at step 3) of the methionine salvage pathway. If this were the case, this block in the salvage pathway would prevent the superfluous and futile demand of downstream proteins such as iron-consuming Adi1 (at step 5). This situation would therefore contribute to limit cellular iron utilization under iron deficiency.

Out of the 137 genes found to be up-regulated by both iron repletion and a *php4*Δ disruption, 119 (87%) of these genes contained one or more copies of the 5′-CCAAT-3′ consensus sequence within their promoters (Table 2). In the cases of *hry1^+^* and *mug14^+^*, a ChIP approach was used to validate that Php4 associated with *hry1^+^* and *mug14^+^* promoters *in vivo*. In the case of genes (13%) lacking the CCAAT consensus sequence, the possibility exists that a noncanonical sequence may act as a functional DNA binding site of the Php2/3/4/5 multimeric complex. Alternatively, an up-regulation of gene expression in the absence of Php4 may be indirect. For example, it is possible that Php4 represses a gene encoding a repressor, which would downregulate expression of a subset of Php4 target genes.

Comparison of the mRNA expression profile of *php4^+^/php4^+^* diploid cells synchronously induced into meiosis under low-iron conditions with cells incubated under iron-replete conditions led to the identification of 57 genes up-regulated after 7 hr of meiotic induction. Some of these genes encoded for known components involved in iron acquisition from inorganic iron and heme (Table S2) (Labbé *et al.* 2013; Mourer *et al.* 2015). We also found several uncharacterized genes, including some that are meiosis-specific (*e.g.*, *spo5^+^*, *cum1^+^*, and *meu3^+^*) for which a putative iron starvation-dependent function remains unclear.

Despite the fact that there was a reduction of *php4^+^* transcripts in iron-replete *fep1^+^*/*fep1^+^* cells, the presence of weak steady-state levels of *php4^+^* mRNA was still detected, revealing an incomplete repression of the transcription of *php4^+^* mRNA. Based on these observations, we expected to detect weak levels of Php4 protein in iron-replete wild-type cells. However, TAP-Php4 steady-state levels were undetectable in iron-replete cells using immunoblot assays. Fluorescent microscopy analysis showed that, 20 min after initiation of the meiotic program in iron-replete cells, GFP-Php4-associated fluorescence disappeared and was not observed during the duration of the meiotic program. mRNA and protein steady-state levels of Php4 exhibited strikingly distinct expression profiles under elevated levels of iron, suggesting the existence of a meiotic posttranslational mechanism that eliminates Php4 or operation of a sequestration system that prevents *php4^+^* transcripts from being translated by the ribosomes. Future experiments are needed to discriminate between these two possibilities. On the one hand, high proteolytic activity occurs in meiotic cells and, on the other hand, nuclear envelope permeability allows atypic transient mixing of nuclear and cytoplasmic proteins during the meiotic program (Arai *et al.* 2010; Asakawa *et al.* 2010; Sazer 2010). The combined effects suggest that the mechanism of iron inhibition of Php4 may be different in dividing cells that grow mitotically as opposed to meiotic cells.

Php4 orthologs in *Candida albicans* (Hap43), *A. fumigatus* (HapX), and *Fusarium oxysporum* (HapX) are required for virulence in mice and plant models of infection (Schrettl *et al.* 2010; Chen *et al.* 2011; Hsu *et al.* 2011; Haas 2012; Lopez-Berges *et al.* 2012). A plausible explanation for these observations is the fact that host organisms offer an iron-poor environment (Becker and Skaar 2014; Ganz and Nemeth 2015), making the iron economy system crucial for the survival of pathogens. In fungi, spores are thought to be infectious particles for many pathogens (Botts and Hull 2010; Oiartzabal-Arano *et al.* 2016). Spores are highly resistant and are adapted for efficient dispersal through airflow or fluids (Geib *et al.* 2016). In order to sporulate, fungal cells have to complete the entire meiotic process that depends on Php4, as shown here for sporulation under low-iron conditions. Because there are several similarities between *S. pombe* and filamentous pathogenic yeasts, the question arises whether Php4 orthologs are also required for meiosis completion in other species. If this were the case, it may be another reason explaining why these proteins are essential for infection.

## 

## Supplementary Material

Supplemental Material
