# Supplementary material for: Php4 Is a Key Player for Iron Economy in Meiotic and Sporulating Cells
Source: G3 (Bethesda). 2016 Jul 26;6(10):3077–95. doi: 10.1534/g3.116.031898 (PMC5068932; doi:10.1534/g3.116.031898)
Supplement: Supplemental Material [file supp_6_10_3077__index.html]

Php4 Is a Key Player for Iron Economy in Meiotic and Sporulating Cells — Supplemental Material 

# Php4 Is a Key Player for Iron Economy in Meiotic and Sporulating Cells

## Supplemental Material for Brault, *et al*, 2016

**Files in this Data Supplement:**

- Table S1 - Genes expressed at higher levels in iron-replete cells. (.xlsx, 26 KB)
- Table S2 - Genes expressed at higher levels in iron-deficient cells. (.xlsx, 15 KB)
- Table S3 - Genes expressed at higher levels in *php4*∆ cells when iron is depleted. (.xlsx, 25 KB)
